# Integrative omics reveals MYCN as a global suppressor of cellular signalling and enables network-based therapeutic target discovery in neuroblastoma

**DOI:** 10.18632/oncotarget.6568

**Published:** 2015-12-11

**Authors:** David J. Duffy, Aleksandar Krstic, Melinda Halasz, Thomas Schwarzl, Dirk Fey, Kristiina Iljin, Jai Prakash Mehta, Kate Killick, Jenny Whilde, Benedetta Turriziani, Saija Haapa-Paananen, Vidal Fey, Matthias Fischer, Frank Westermann, Kai-Oliver Henrich, Steffen Bannert, Desmond G. Higgins, Walter Kolch

**Affiliations:** ^1^ Systems Biology Ireland, University College Dublin, Belfield, Dublin, Ireland; ^2^ Conway Institute of Biomolecular & Biomedical Research, University College Dublin, Belfield, Dublin, Ireland; ^3^ School of Medicine and Medical Science, University College Dublin, Belfield, Dublin, Ireland; ^4^ Division of NB Genomics, German Cancer Research Center (DKFZ), Heidelberg, Germany; ^5^ Department of Paediatric Haematology and Oncology and Center for Molecular Medicine Cologne (CMMC), University Hospital Cologne, Cologne, Germany; ^6^ VTT Technical Research Centre of Finland, Tietotie 2, Espoo, Finland; ^7^ The Whitney Laboratory for Marine Bioscience, University of Florida, St. Augustine, Florida, USA; ^8^ European Molecular Biology Laboratory (EMBL), MeyerhofstraΔe, Heidelberg, Germany

**Keywords:** MYC (c-MYC), neuroblastoma, transcriptional regulation, mRNA sequencing (mRNA-seq), 4sU-seq

## Abstract

Despite intensive study, many mysteries remain about the MYCN oncogene's functions. Here we focus on MYCN's role in neuroblastoma, the most common extracranial childhood cancer. MYCN gene amplification occurs in 20% of cases, but other recurrent somatic mutations are rare. This scarcity of tractable targets has hampered efforts to develop new therapeutic options. We employed a multi-level omics approach to examine MYCN functioning and identify novel therapeutic targets for this largely un-druggable oncogene. We used systems medicine based computational network reconstruction and analysis to integrate a range of omic techniques: sequencing-based transcriptomics, genome-wide chromatin immunoprecipitation, siRNA screening and interaction proteomics, revealing that MYCN controls highly connected networks, with MYCN primarily supressing the activity of network components. MYCN's oncogenic functions are likely independent of its classical heterodimerisation partner, MAX. In particular, MYCN controls its own protein interaction network by transcriptionally regulating its binding partners.

Our network-based approach identified vulnerable therapeutically targetable nodes that function as critical regulators or effectors of MYCN in neuroblastoma. These were validated by siRNA knockdown screens, functional studies and patient data. We identified β-estradiol and MAPK/ERK as having functional cross-talk with MYCN and being novel targetable vulnerabilities of MYCN-amplified neuroblastoma. These results reveal surprising differences between the functioning of endogenous, overexpressed and amplified MYCN, and rationalise how different MYCN dosages can orchestrate cell fate decisions and cancerous outcomes. Importantly, this work describes a systems-level approach to systematically uncovering network based vulnerabilities and therapeutic targets for multifactorial diseases by integrating disparate omic data types.

## INTRODUCTION

Since its discovery over 30 years ago in the childhood cancer neuroblastoma [1], MYCN has been implicated in a wide array of developmental and cancer-associated processes [2-12]. The oncogenic potential of the MYC gene family is ancient, and conserved throughout the metazoan lineage [13, 14], but the extent to which MYCN and the more widely expressed c-MYC (MYC) are just tissue-specific homologues or fulfil non-redundant functions is still unclear. Their protein sequences substantially diverge, and they are spatially and temporally differentially expressed (DE) [15, 16]. Yet, they share many target genes, and Mycn, inserted into the c-Myc locus, can rescue the embryonic lethality of a c-Myc knockout mouse [2]. However, a Mycn but not c-Myc conditional knockout can prevent Sonic hedgehog (SHH) induced medulloblastoma precursor cell proliferation *in vitro* and medulloblastoma formation *in vivo* [3].

MYCN has been linked to numerous cancers [17, 18] and MYCN amplification status is the strongest single gene predictor of prognosis in neuroblastoma [4-7, 11]. About 20% of neuroblastoma patients have MYCN amplified (MNA) tumours, and are prone to treatment resistance, relapse, development of metastases and low survival [11]. Neuroblastoma accounts for the highest proportion of all childhood cancer deaths [19], on account of this MNA patient cohort. Yet, a complete picture of oncogenic MYCN function or a conceptual integration of its different molecular roles is still absent. This is partly due to MYCN being a broadly acting, but weak transcription factor [20], and because it has other functions that include global interactions with the epigenetic and chromatin remodelling machineries [21, 22]. Therapeutic approaches to tackle high-risk MYCN-amplified neuroblastoma are lacking, and a disproportionate number of high-risk individuals die or suffer treatment related morbidity [23]. While some progress appears to be on the horizon [24-26] new therapeutic approaches need to be identified.

**Figure 1:**
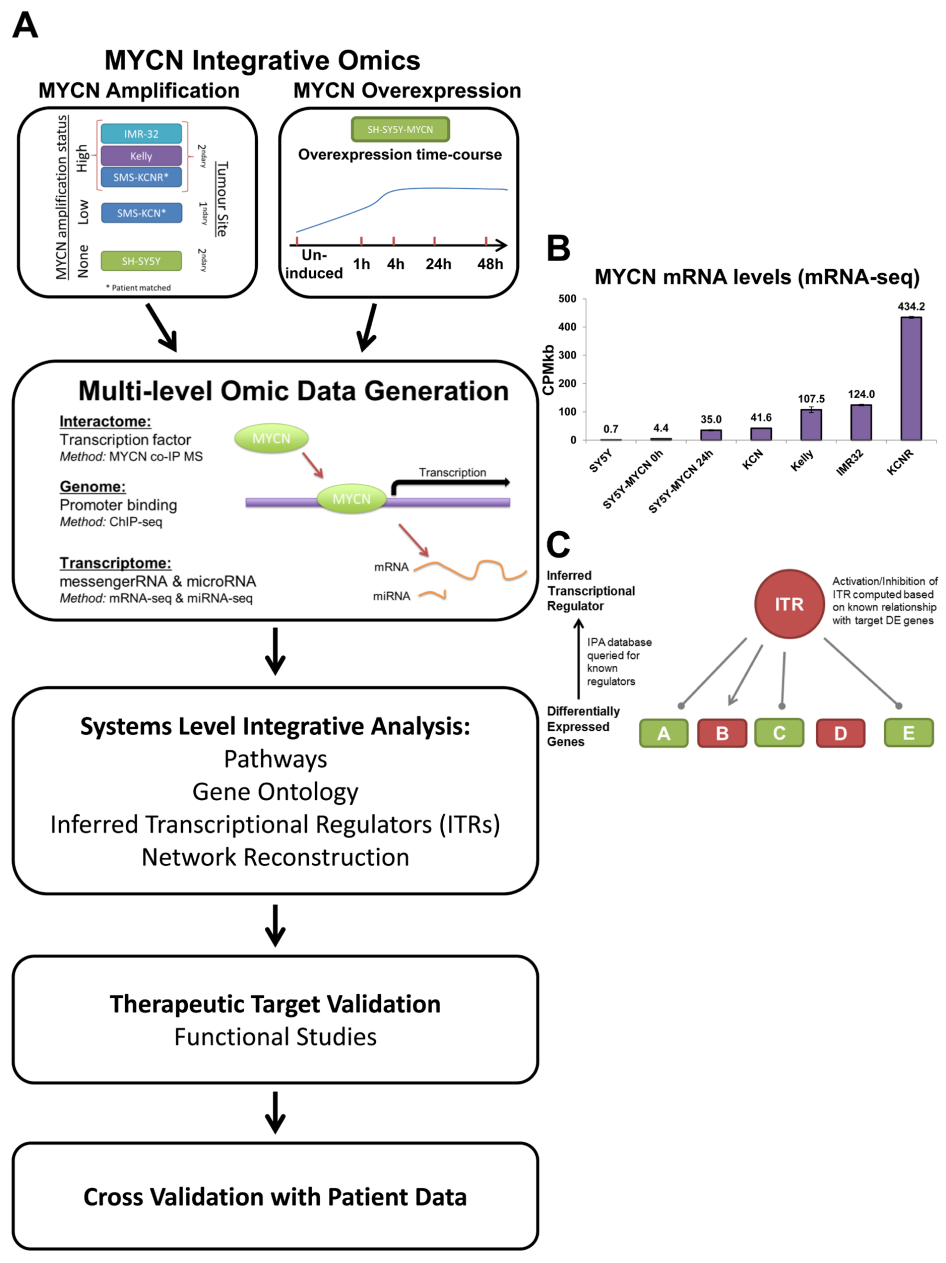
An integrative omics approach for analysing MYCN networks **A.** Schematic outline of the experimental and analysis approach. **B.** MYCN mRNA expression in each mRNA-seq sample. CPMkb: counts per million adjusted by gene length in kilobases. Single-read runs are denoted by (sr), with the remainder of the samples being generated by paired-end runs. The time after the SY5Y-MYCN samples indicates the duration of MYCN induction, with 0h being un-induced. **C.** Schematic overview of how the inferred transcriptional regulators (ITRs) of a set of differentially expressed (DE) genes are identified by Ingenuity Pathway Analysis (IPA). Here, transcription of genes A, C and E is inhibited by the ITR, while B is activated by it, and D is DE but not regulated by the ITR.

The paucity of recurrent somatic mutations in neuroblastoma has hampered classical genetic approaches, which rely on frequently altered oncogenic drivers, to identify novel targets for the treatment of high-risk neuroblastoma [27]. To overcome this obstacle we have taken a network-based approach to elucidate novel therapeutic targets for MNA neuroblastoma, which focuses on the functional status of downstream biological networks rather than the heterogeneous upstream mutation and epigenetic events. In order to map these networks we took an unbiased integrative omics approach combining transcriptomic analysis of MYCN target genes (4sU-seq, mRNA-seq and miRNA-seq) with MYCN DNA-binding analysis (ChIP-seq) and proteomic identification of MYCN-binding proteins (mass spectrometry) under different conditions (Figure 1A). To investigate how MYCN regulates its downstream effector networks we took a network centred approach, where we analysed the effects of MYCN on different types of molecular networks and then connected them via shared functionalities. The resulting networks were then validated using drug treatments of cell lines, a druggable-genome RNAi knockdown screen, and neuroblastoma patient data. Of the analysis approaches utilised on the omic data we found that integration at the level of transcriptional regulators provided the best framework for both identifying novel therapeutic targets and stratification of patients.

## RESULTS

### Integration of overexpressed and amplified MYCN omics

Although important for the genesis of neuroblastoma [28, 29], MYCN overexpression without gene amplification is often associated with good outcomes [8-10]. However, MNA is the strongest predictor of poor clinical outcome [4-7, 11]. Therefore, we compared MYCN overexpressing and amplified MYCN cells. By applying a variety of omics technologies and systems level analysis approaches, we investigated the global functioning of the MYCN oncogene in both an overexpressed and amplified setting (Figure 1A). Overexpressed MYCN was examined in the MYCN inducible cell line SY5Y-MYCN. As due to the heterogeneous nature of the amplification process, no single representative MNA model exists, we also used a panel of neuroblastoma cell lines which broadly represent the range of neuroblastoma, from MYCN single copy to amplified, and which cover a wide range of MYCN expression (Figure 1B).

We found that integration of the data at the level of transcriptional regulators provided the best framework for identifying novel therapeutic targets for further validation. The Ingenuity Pathway Analysis (IPA) suite was used to identify the inferred transcriptional regulators (ITRs) of differentially expressed (DE) genes (Figure 1C). This analysis uses statistical algorithms to match observed DE genes to known gene regulatory modules from the curated IPA knowledge database, thus inferring the regulator(s) of a set of genes based on the known patterns of genes regulated by a specific gene, protein or chemical compound [30]. This analysis also extracts information about the activation status of the ITR molecules, based on the concept that downstream transcriptional target activity reflects the activation status of their upstream regulators [30] (Figure 1C).

### Integrative omics of MYCN reveals a function as a transcriptional repressor

**Figure 2:**
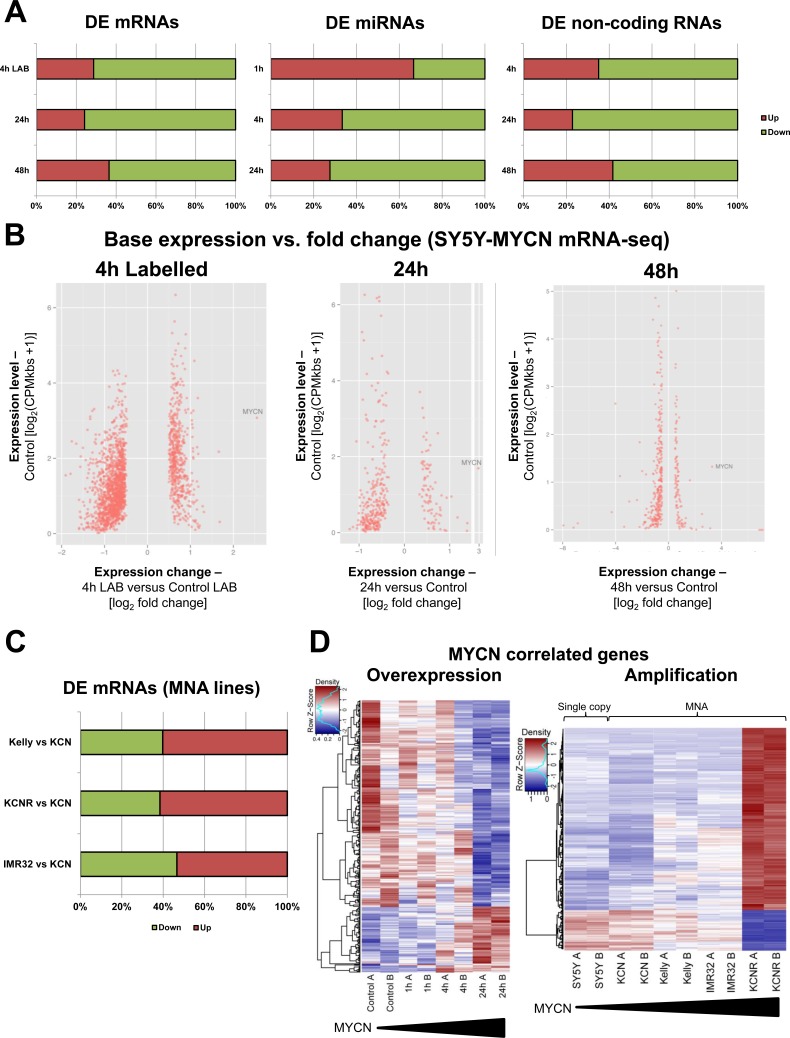
MYCN can repress or activate gene expression dependent on MYCN gene amplification status **A.** Proportions of up- and down-regulated mRNAs (mRNA-seq), miRNAs (miRNA-seq) and non-coding RNAs (mRNA-seq) upon MYCN overexpression (SY5Y-MYCN). All data are normalised to relevant control un-induced samples. The 4h LAB time-point is from 4sU labelled cells. **B.** All significant differentially expressed (DE) genes, with expression level in the un-induced state plotted against the fold change after MYCN induction. 4h Labelled, 24h and 48h MYCN overexpression time-points shown. Each significant DE gene is denoted by a red dot. **C.** Proportions of up- and down-regulated mRNAs (mRNA-seq), pair-wise comparisons between each of the metastatic MNA lines and KCN (edgeR). **D.** Heat maps of the MYCN overexpression time-course (as compared by edgeR) and the cell line MYCN correlated genes (as compared by DESeq), from mRNA-seq. A, B denote the biological duplicates. Samples ordered by MYCN expression level.

MYCN overexpression predominantly repressed target gene expression at the levels of mRNA, miRNA (4h and 24h after MYCN induction) and non-protein coding RNA (Figure 2A). MYCN expression levels rapidly increased after induction in the SY5Y-MYCN line (Figure S1A). RNA-seq results were validated by qPCR for selected targets (Figure S1B). In addition to standard mRNA-seq where total mRNA is profiled, we employed 4sU pulse labelling of newly-transcribed mRNA (label added for the last 30min before extraction), for the un-induced, 1h and 4h MYCN overexpression time-points, for an in-depth description of the experiment see Schwarzl et al. [31]. The enrichment of actively transcribed genes (Figure S1C), achieved by 4sU pulse labelling of newly-transcribed mRNA, enabled the identification of previously undetectable early stage MYCN targets (Figure S1D). Importantly, MYCN mediated gene regulation did not just amplify the previous expression state of a gene, but acted independently of the gene's initial expression level (Figure 2B).

Pair-wise comparison of the MNA lines showed that the number of upregulated genes tended to increase with higher MNA levels (Figure 2C). In contrast to overexpression, MYCN amplification predominantly upregulated targets (Figure 2D) with over four times more genes positively than negatively correlating to MYCN expression. The genes regulated by amplified and overexpressed MYCN were largely different (Figure S1E), indicating a switch in targets rather than differential regulation of the same genes. Thus, MYCN's effect on target gene expression is fundamentally different depending on whether it is overexpressed (repressor) or amplified (activator).

### MYCN preferentially binds to gene enhancers and its oncogenic functions are largely independent of its canonical binding partner MAX

**Figure 3:**
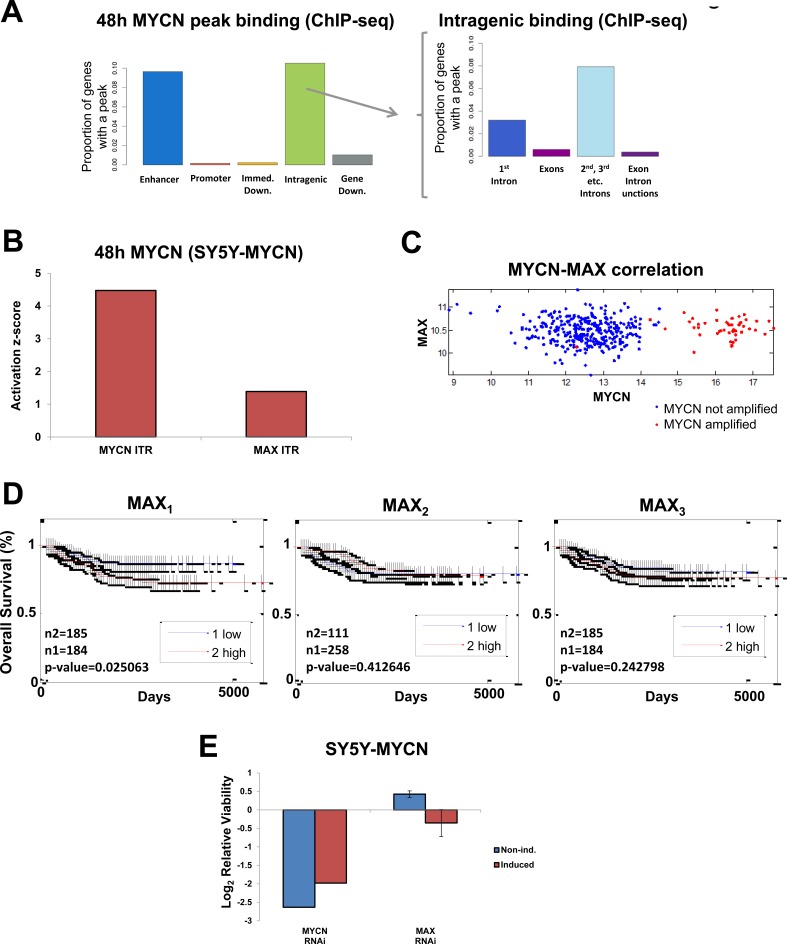
Global MYCN DNA binding profile and MYCN-MAX divergent functions **A.** MYCN genome binding (ChIP-seq) is predominantly to enhancers (left) and intragenic regions (right). Abbreviations: Immed. = immediate, Down. = downstream. **B.** MYCN and MAX ITR activation at 48h of MYCN induction, as revealed by IPA analysis of the mRNA-seq data. Activation status is based on differential regulation of known target genes. **C.** MYCN and MAX mRNA levels are not correlated with each other in the patient data (microarray). **D.** MAX mRNA expression levels measured by microarray do not correlate to neuroblastoma patient outcome. Three MAX probes shown (MAX_1-3_). **E.** RNAi screen viability results for MYCN and MAX knockdown in SY5Y-MYCN cells. In the induced samples MYCN overexpression was activated 24h prior to and maintained throughout the subsequent 72h RNAi treatment.

Both overexpressed and amplified MYCN predominantly bound to gene enhancers (ChIP-seq), while binding to gene body regions showed a clear preference for introns (Figure 3A). As expected the RNAPolII ChIP-seq control showed preferential binding to promotors (Figure S2A). MYCN bound to many transcriptional targets within gene bodies, matching recently reported gene body methylation sites [32], suggesting that MYCN may be directing the subsequent methylation of its targets. Previous global MYCN genome binding assays generally used ChIP-chip with promoter enriched microarrays, therefore missing the enhancer binding detected by our unbiased ChIP-seq approach. Similar to our human MYCN analysis, a previous study of MYCN genome binding in mice which also used ChIP-seq revealed that only 33% of MYCN binding peaks were located within promoter regions [33] (see www.cistrome.org database).

We found evidence that oncogenic MYCN regulates gene expression independent of the classic c-MYC and MYCN heterodimer partner MAX. There was a lack of E-box binding upon MYCN overexpression or amplification (MYCN ChIP-seq) according to SIOMICS motif finder analysis [34] (data not shown). Similarly, MAX was not a top transcription factor associated with overexpressed and amplified MYCN's genomic targets using the DiRE [35] tool, with a very small proportion of the MYCN bound genes having known MAX regulatory elements (Figure S2B). Supporting this interpretation, MAX mRNA expression levels were consistent across all cell lines tested regardless of their MNA status or the level of MYCN expressed (Figure S2C), while MXD1 and MXI1 tended to increase with higher MYCN expression (Figure S2D). MXD1 and MXI1 both sequester MAX [36, 37], preventing MYCN binding. Consistent with this, when the DE genes were analysed for their known transcriptional regulators using IPAs' inferred transcriptional regulators (ITRs) algorithm [30], MYCN activation outstripped MAX activation levels (Figure 3B). In addition, when highly MNA cells (KCNR) were compared with MYCN single copy cells (SY5Y), MAX was not an ITR of the DE genes.

We next assessed these findings in a well-characterised neuroblastoma tumour microarray dataset comprising 478 patients [38], where we found no correlation between MAX and MYCN mRNA levels (6, 10], there was no strong correlation between MAX expression and overall patient survival (40, 41]. Therefore, binding partners other than MAX are likely more relevant to driving MYCN-mediated poor neuroblastoma outcome.

### MYCN transcriptionally regulates its own interaction partners

**Figure 4:**
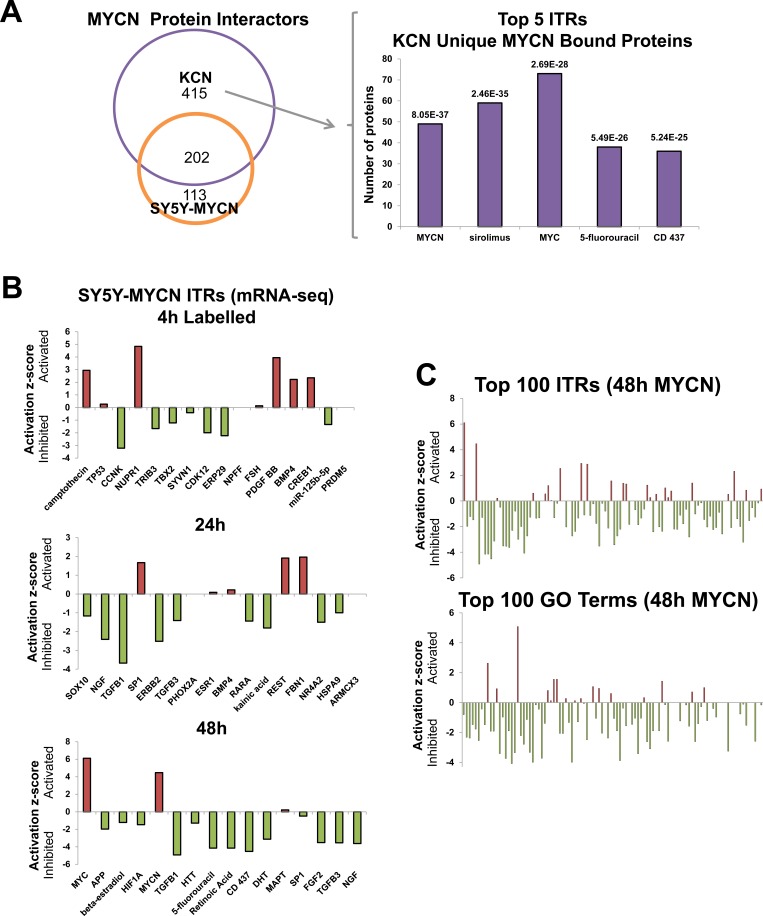
MYCN protein-protein interactors, and global repression of cellular networks by overexpressed MYCN **A.** Overlap between MYCN binding proteins, as assessed by CoIP-MS analysis, in MYCN amplified KCN and 24h overexpressed MYCN in SY5Y-MYCN (left). The top 5 ITRs, identified with IPA, of the 415 proteins which only bound amplified MYCN, KCN cells but not overexpressed MYCN (SY5Y-MYCN) (right). Number of the proteins regulated per ITR shown on the y-axis and the p-value of overlap with all known genes associated with that ITR shown above each bar. **B.** Activation/inhibition z-scores of the top ITRs (IPA) of the genes differentially expressed at 48h, 24h and 4h LAB (4sU labelled) of MYCN overexpression. **C.** Global view of the activation/inhibition z-scores of the top 100 ITRs (top) and disease and functions GO terms (bottom) for 48h MYCN overexpression (IPA). Ranked by p-value of overlap, of the number of ITR/term component genes which were differentially expressed. GO terms and ITR names are shown in Table S2, the majority of both were downregulated.

To determine binding partners other than MAX which could contribute to oncogenic MYCN functions we next evaluated proteins which differentially bind to MYCN protein in an amplified (KCN) versus an overexpressed (SY5Y-MYCN) setting using quantitative interaction proteomics. MYCN mRNA and protein expression levels in KCN are comparable to 24h induced SY5Y-MYCN (Figure S3A). While most MYCN-interacting proteins bound to both overexpressed and amplified MYCN, 415 proteins uniquely bound to amplified MYCN (Figure 4A). When these 415 proteins were analysed for their known transcriptional regulators, by ITR analysis, MYCN was the top ITR, and c-MYC (the MYCs share many redundant functions and targets [2]) was third (Figure 4A). Thus, MYCN is capable of re-wiring its own interactome through the transcriptional regulation of its interaction partners, which likely contributes to the differences between overexpressed and amplified MYCN functions. The other 3 molecules in the top 5 ITRs were drugs (Figure 4A), i.e. sirolimus (rapamycin) targeting mTORC1, which inhibits neuroblastoma proliferation [42]; 5-Fluoro-Uracil (5-FU), an inhibitor of DNA synthesis used in cancer therapy including neuroblastoma [43]; and CD437, a retinoid acid analogue that induces apoptosis in neuroblastoma [44]. These results indicate an intriguing overlap of amplified MYCN function with drugs that are used in cancer therapy and regulate cell growth (rapamycin), DNA synthesis (5-FU), and apoptosis (CD437).

### Response kinetics of overexpressed MYCN target genes are temporally dynamic, but regulatory mechanisms are conserved

To elucidate how MYCN alters cell fate, we next applied a systems level analysis to examine global network properties of overexpressed and amplified MYCN. The response of individual genes differentially expressed upon MYCN overexpression was highly temporally dynamic as seen at the DNA binding, mRNA and miRNA levels (Figure S4A-C). Therefore, to better extract meaning from both the time-course and the various omic platforms we integrated the datasets at a systems level, using pathway and gene ontology (GO) analysis, network reconstruction, and inferring the transcriptional regulators. This integration revealed a much higher degree of overlap than gene level integration, both in the temporal and the cross-platform data (Figure S4D). Similarly, the MYCN interacting proteins remained rather consistent across the time-course (Figure S4E) suggesting that overexpressed MYCN functional changes are mediated by a similar set of MYCN interacting proteins despite the temporally dynamic response of target genes.

Given that the protein interactors of overexpressed MYCN were largely temporally consistent (Figure S4E), but that amplified MYCN bound to many proteins not bound by overexpressed MYCN (Figure 4A) we next assessed whether the transcriptional regulators driving differential gene expression were different between MYCN overexpression and amplification. ITR analysis of the various RNA-seq samples revealed that overexpressed MYCN (48h) and amplified MYCN share a core set of transcriptional regulators accounting for approximately 40% of their top 100 transcriptional regulators (Table 1). However, beyond these core regulators overexpressed and amplified MYCN functioning diverged, with over 70% of the ITRs (of the top 100 ITRs in each MNA line compared to MYCN single copy cells) being common to the MNA cell lines (Table 1). Interestingly, there was 71% overlap between the MNA cell lines with the lowest and highest level of MYCN expression, but only 43% overlap between the matched MYCN expression (Figure S3A) cell lines KCN and 48h overexpression SY5Y-MYCN (Table 1), showing that the differing levels of overlap are not due to dosage effects. Therefore, there exists a large cohort of proteins which bind exclusively to amplified MYCN (Figure 4A), which are likely responsible for the diverging transcriptional regulation seen between overexpressed and amplified MYCN.

**Table 1:** Percentage overlaps of the top 100 ITRs from each RNA-seq cell line

	48h MYCN	KCN	Kelly	IMR32	KCNR
48h MYCN	100	43	42	41	43
KCN	43	100	73	74	71
Kelly	42	73	100	74	70
IMR32	41	74	74	100	78
KCNR	43	71	70	78	100

*denotes Overexpression Vs amplification comparisons highlighted in brown. ^denotes Amplification Vs amplification comparisons highlighted in blue.

Among the amplified and overexpressed MYCN's conserved effects was regulation of the axonal guidance signalling pathway, which was the top ranked pathway at both 24h and 48h of MYCN overexpression, and between each of the MNA lines and SY5Y (Table S1). In addition to their role in neuronal development, axonal guidance genes are also involved in tumourigenesis [45]. Interestingly, many components of sonic hedgehog (SHH) signalling were downregulated by 4h (Figure S5A). The expression of SHH pathway proteins is suppressed in tumours of MNA patients [46], suggesting that inhibition of SHH signalling contributes to MYCN initiated transformation.

### Both overexpressed and amplified MYCN primarily supress cellular networks

We next examined the effect of amplified and overexpressed MYCN on global cellular signalling networks, using ITR and GO term analysis. This revealed that the majority of early time-point DE genes we identified are novel MYCN targets, as c-MYC and MYCN were only top ITRs at 48h (Figure 4B). Mirroring the gene expression level, MYCN overexpression predominantly inhibited ITRs and GO terms (Figure 4C, Table S2). Although overexpressed MYCN controls a diverse array of genes and functions, the core ITRs common in at least 2 time-points, form a highly connected regulatory network (Figure S5B, Table S3). This network consists of both known (e.g. NTRKs, TGFβ, p53, BRDs and RXRs) and novel (e.g. NUPRs, HIFs and MAPKs) MYCN interactors and neuroblastoma regulators.

Similar to MYCN overexpression, the MNA lines showed a high degree of overlap between the top ITRs (Table S4) and pathways of each line (Figure S6A). Again, the majority of ITRs (Figure S6B, including 14 of the top 15) and GO terms were inhibited with increasing MNA levels. This suggests that despite the differences in gene level regulation by overexpressed and amplified MYCN, both MYCN states primarily function through the repression of cellular networks, suggesting that MNA enhances oncogenesis predominantly through suppressing signalling pathways and transcriptional regulators. Genes from numerous cancer pathways were differentially expressed not only between MYCN single copy and MNA cell lines, but also between the patient-matched MNA lines KCN and KCNR (Figure S7A).

We further assessed how overexpressed and amplified MYCN differ in their regulation of cell fate. Comparing 48h MYCN overexpression (SY5Y-MCYN) with amplification (KCNR) revealed that, while some similarities exist between overexpressed and amplified MYCN functions, there are considerable differences (Figure S7B). Generally, the GO terms common to both lines and those unique to SY5Y-MYCN tended to be weighted more heavily towards normal processes, while the KCNR unique terms were more heavily weighted towards disease (Figure S7B), supporting the hypothesised link between MYCN dosage and molecular oncogenic functioning.

### Network analysis reveals therapeutically targetable nodes: MAPK/ERK pathway

**Figure 5:**
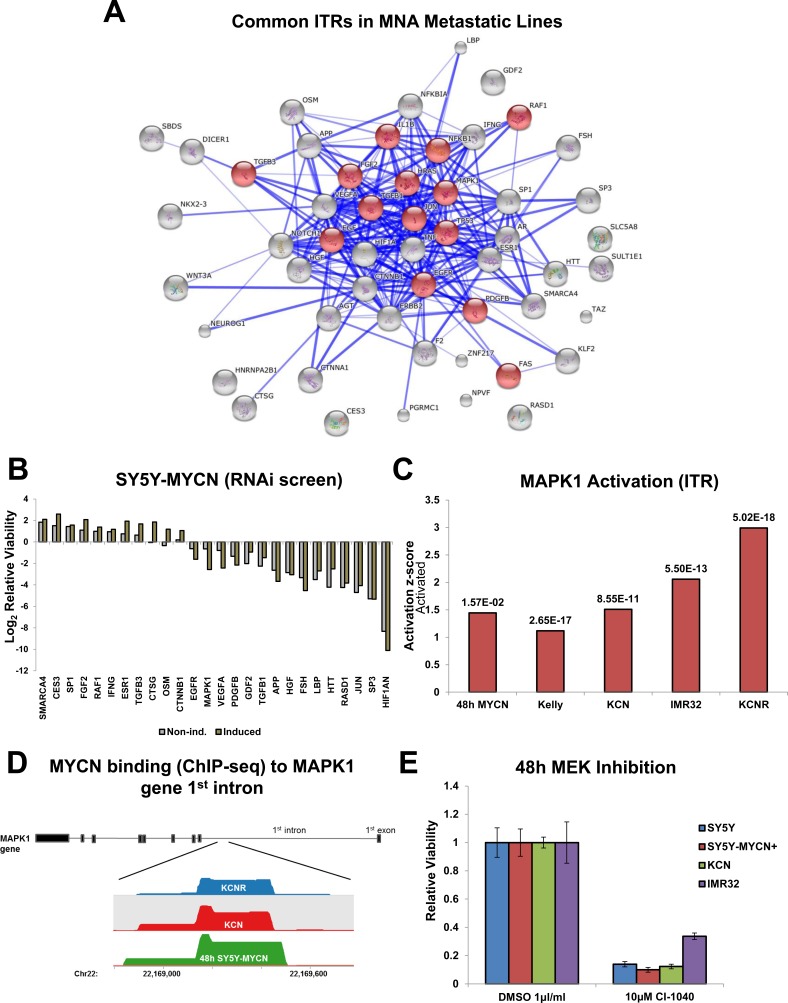
Identification of therapeutically targetable nodes through ITR and network analysis: MAPK **A.** The 53 common ITRs (from top 100) between the 3 metastatic MNA lines (IMR32, Kelly, KCNR) were identified. Then a protein interaction map of previously known connections between them was generated with String. MAPK associated nodes, as defined by String's KEGG pathway enrichment tool, are shaded red. **B.** 72h RNAi screen viability results for the genes from the MNA protein interaction map which increased or reduced cell viability by at least +/− 1 log_2_FC. In induced samples MYCN overexpression was induced 24h prior to and maintained throughout the RNAi treatment. **C.** Activation z-scores of MAPK1 in the mRNA-seq samples where it was an ITR, p-value of overlap (between the total number of known MAPK1 ITR component genes and those which were differentially expressed) shown above each bar. **D.** MYCN binding peaks, located in the 1^st^ intron of the MAPK1 gene, as detected by MYCN ChIP-seq in the SY5Y-MYCN (48h of MYCN overexpression), KCN and KCNR cell lines. Track visualisation was performed using SeqMonk. **E.** Relative viability of neuroblastoma cells treated with the MEK inhibitor CI-1040, as assed by MTS assay and relative to control cell viability. SY5Y-MYCN+ indicates that MYCN expression was induced for 72 hours.

Given the findings that oncogenic MYCN functioning occurs primarily independent of MAX signalling and that regulatory differences exist between overexpressed and amplified MYCN, we next used our MNA network and ITR analysis to identify oncogenic MYCN co-regulators which represent therapeutically targetable nodes. Of the top 100 ITRs per cell line 62 (Table S5) were common to all 3 metastatic MNA lines (IMR32, Kelly and KCNR). When the protein ITRs were connected based on known protein-protein interactions (drug ITRs excluded), they formed a highly interconnected interaction network (Figure 5A), which was significantly (p=4.210E-9) enriched for MAPK (ERK) signalling (14 of 53 components) based on String's KEGG pathway enrichment tool. Of the components of this network which were present in the druggable-genome siRNA screen, 21 strongly (by at least +/−1 log2FC) altered cell viability when knocked down (Figure 5B, Table S5). The MYCN overexpression ITR network (ITRs present in at least two RNA-seq time-points, (Figure S8A) was also enriched for MAPK signalling genes (String's KEGG pathway enrichment tool, p=1.35E-13). In addition, amplified MYCN proteins physically interacted with 19 components of the MAPK/ERK signalling pathway in KCN cells (IPA, p-value of overlap = 6.46E-06). None of these MAPK component proteins have previously known interactions with MYCN (Figure S8B).

We therefore examined MAPK ITRs present in the RNA-seq analysis. Of these MAPK1 (ERK2) was the highest ranked ITR and its activation further increased upon both MYCN overexpression and amplification (Figure 5C). Intriguingly, the MYCN ChIP-seq data revealed that MYCN bound to the first intron of the MAPK1 gene in all cell lines tested (Figure 5D). Additionally, MAP2K1 (MEK1), the direct regulator of MAPK1, was one of the novel protein-protein interactors of amplified MYCN (Figure S8B). Interestingly, MAPK1 knockdown reduced SY5Y-MYCN cell viability in a MYCN dosage dependent manner (Figure 5B). ERK pathway activation has been previously observed in NF1 mutated neuroblastoma [47], but not previously found correlated with MYCN expression levels. All neuroblastoma lines tested showed reduced viability upon ERK pathway inhibition using the MEK inhibitor CI-1040 (PD184352) (Figure 5E). Therefore, our findings reveal a number of novel points of functional cross-talk between MYCN and the MAPK signalling pathway, suggesting that ERK inhibition could be a viable target not only for NF1 mutated, but neuroblastoma in general including MNA neuroblastoma.

### Network analysis reveals therapeutically targetable nodes: β-estradiol

**Figure 6:**
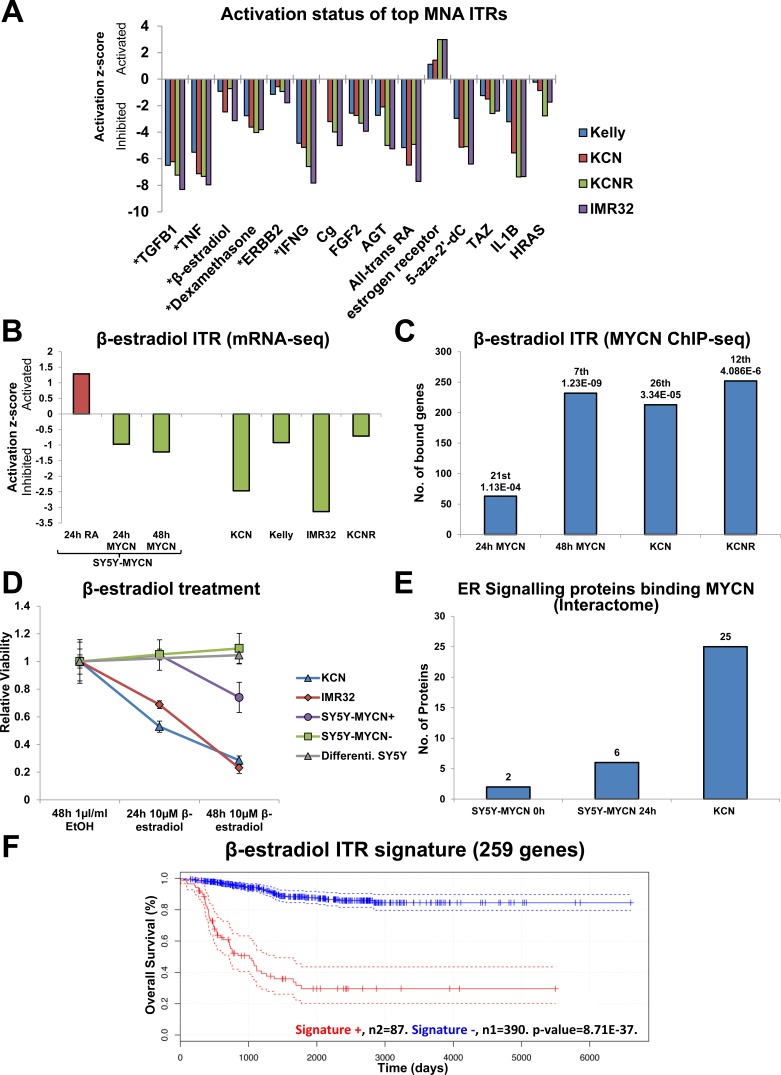
Identification of therapeutically targetable nodes through ITR and network analysis: β-estradiol **A.** Activation/inhibition z-score plot of the top 10 ITRs (IPA) for each of the 4 MNA cell lines compared with SY5Y (mRNA-seq). * denotes regulators that were in the top 10 ITRs for all lines. **B.** Inhibition z-scores of β-estradiol ITR in the MNA (versus SY5Y), Retinoic Acid (RA) treated (un-induced) SY5Y-MYCN and MYCN overexpressing SY5Y-MYCN (both *versus* un-induced SY5Y-MYCN) mRNA-seq samples. **C.** Number of MYCN bound genes (ChIP-seq) that are also known β-estradiol targets according to IPA analysis. Rank in ITR analysis and p-value of overlap (between the bound genes and all known β-estradiol targets) shown above each bar. **D.** Viability of neuroblastoma cells relative to control cells when treated with β-estradiol, as measured by MTS assay. SY5Y-MYCN+ overexpressed MYCN, while SY5Y-MYCN- were un-induced. SY5Y Differenti. Are SY5Y cells pre-treated with 1μM RA for 8 days to induce differentiation into neurons, they were also cultured in the presence of RA for the duration of the 48h β-estradiol treatment. **E.** The number of ER signalling associated proteins which bound the MYCN protein (CoIP) in SY5Y-MYCN, MYCN un-induced (0h) and overexpressing (24h) and MNA KCN. **F.** The DE genes contributing to the identification of β-estradiol as an ITR of the mRNA-seq data was used to generate a gene signature. Only genes which were DE in at least 2 cell lines were used. This signature for β-estradiol (bottom) was predictive of neuroblastoma patient outcome.

Interestingly, the ITR analysis of the MNA cell lines revealed that despite them being phenotypically diverse they share common regulatory mechanisms, with a remarkable degree of similarity between the top ITRs and their extent of activation/inhibition in each MNA line compared to SY5Y (Figure 6A). TGFB1 was the top ITR in each of the four MNA lines, and was strongly inhibited. MYCN has previously been shown to repress TGFβ signalling through the activation of miR-17-92 [48]. As the ITR analysis can also include chemical compounds as regulators, we employed it to identify the top chemical regulators associated with neuroblastoma (Figure 6A). The chemical ITRs showing inhibition in MNA cells versus MYCN single copy cells present candidate therapeutics for MNA neuroblastoma, as treating MNA cells with these drugs could conceivably compromise the detrimental effects of MYCN. ITR analysis correctly identified all-trans Retinoic Acid (RA, Tretinoin), and 5-aza-2′-dC (Decitabine). RA is used clinically to treat neuroblastoma [49], and Decitabine is currently in clinical trials for neuroblastoma. These results provided confidence in the ITR analysis and prompted us to assess the top chemical ITR, β-estradiol.

β-estradiol was inhibited across the MNA lines and upon MYCN overexpression (Figure 6A, 6B). Conversely, β-estradiol is activated when SY5Y-MYCN cells are induced to differentiate with RA (Figure 6B). β-estradiol was also a top ITR when considering genes bound by overexpressed MYCN in SY5Y-MYCN and amplified MYCN in KCN and KCNR cells (Figure 6C). Interestingly, MYCN overexpression or amplification conferred sensitivity to β-estradiol treatment, resulting in reduced neuroblastoma cell viability (Figure 6D), although the extent of β-estradiol ITR inhibition did not correlate solely with amplified MYCN expression (Figure 6B). Un-induced SY5Y-MYCN cells and parental SY5Y cells induced to differentiate into neurons with a 8 day RA pre-treatment were completely resistant to β-estradiol treatment (Figure 6D), indicating that this could be a promising MYCN targeted therapy producing little off-target neuronal damage. The canonical receptors for β-estradiol are the Estrogen receptors (ERs). ER signalling was also an ITR of all overexpressed MYCN ChIP-seq time-points and amplified MYCN (Figure S9A). The ER pathway was also enriched in amplified MYCN specific protein interactors (Figure S9B). While the number of ER pathway proteins binding MYCN protein increased somewhat upon overexpression, amplified MYCN showed a vast increase (Figure 6E). Thus, the sensitivity of neuroblastoma cell lines to β-estradiol may correlate with the proteins interacting specifically with MYCN.

To assess whether these results were more generally applicable to neuroblastoma tumour biology we examined the cell line ITR analysis results for β-estradiol in the well-characterised neuroblastoma tumour microarray dataset comprising 478 patients [38]. The activity of the β-estradiol ITR was inhibited in MNA cell lines, and 24h and 48h MYCN overexpression. A gene signature was generated using the DE genes upon which the inhibition of this ITR was based (Table S6). Only genes that were DE in at least two cell lines were used. The β-estradiol ITR signatures showed predictive ability, correctly segregating patient survival cohorts (Figure 6F). Therefore, our systems level analysis not only identifies novel therapeutic targets, but also simultaneously identifies specific signatures for each target which could be used to potentially stratify patients into treatment groups.

Further supporting the likelihood of their therapeutic potential β-estradiol and MAPK1 were all ITRs of the 674 genes that strongly reduced SY5Y-MYCN cell viability when knocked down in the high-throughput RNAi screen (Figure S9C). Three of the top four ITRs from this RNAi screen (TNF, TGFB1 and β-estradiol) were also in the top four ITRs of the MYCN amplified versus single copy cell analysis (Figure 6A), revealing a remarkable ability of ITR analysis to identify relevant regulators of neuroblastoma even from highly disparate experimental approaches.

### Differentiation therapy sensitises MNA cells to the therapeutic targeting of the MYCN oncogenic network

**Figure 7:**
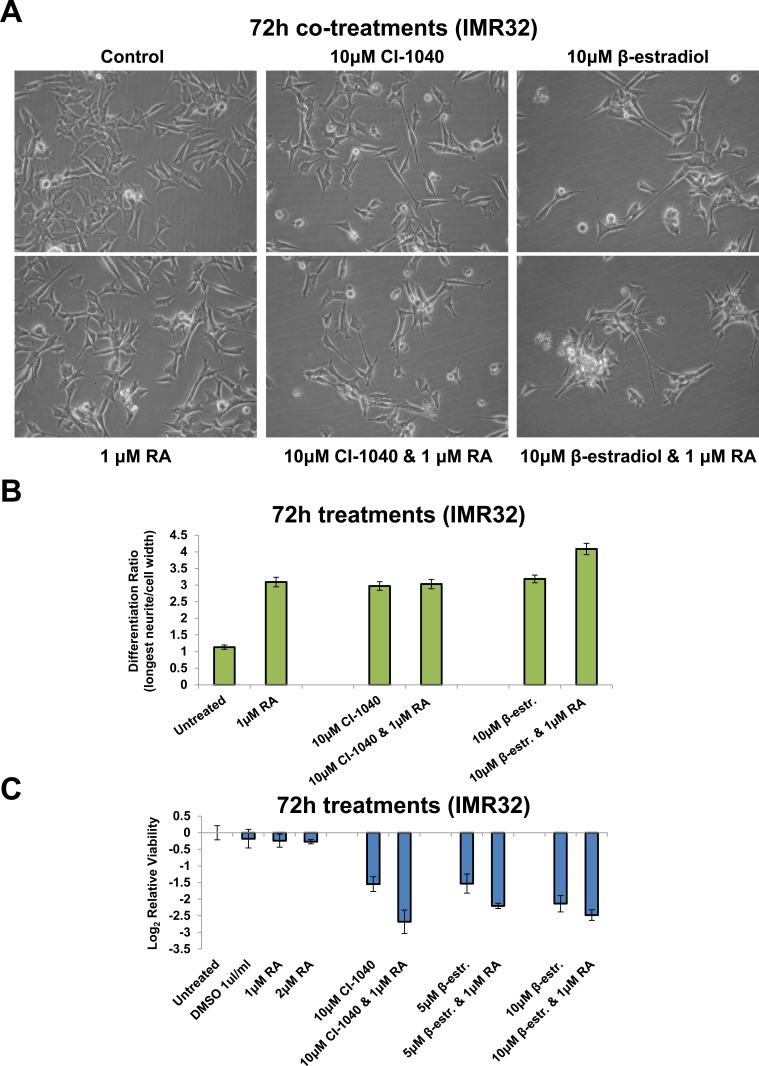
Retinoic Acid, CI-1040 and β-estradiol combination treatments **A.** Imaging of IMR32 cells treated for 72h with MAPK inhibitor (CI-1040) or β-estradiol singly, or in combination with Retinoic Acid (RA). All panels are imaged at 40x magnification. **B.** The differentiation ratio of IMR32 cells treated for 72h with individual agents or combination treatments with RA, was calculated by dividing the length of the longest neurite of a cell by the cell's width. Measurements made using ImageJ v1.44p (http://imagej.nih.gov/ij). Range of measured cells (N) per treatment group is 132-209. Error bars depict the standard error of the mean. **C.** Cell viability analysis of IMR32 cells treated for 72h with individual agents or combination treatments with RA, as detected by MTS assay.

MYCN amplification inhibits neuroblastoma differentiation [50, 51]. Our RNA-seq GO term analysis confirmed that MNA cells were less differentiated in terms of neuritogenesis than MYCN single-copy SY5Y (Figure S10A). Having identified the regulator networks through which amplified MYCN orchestrates its effects, we next assessed the phenotypic effect of perturbing network components. MAPK (MEK) inhibition (CI-1040) in the MNA cell line IMR32, not only reduced cell viability, but surviving cells became more differentiated in appearance, with longer neurite outgrowths and narrower cell bodies (Figures 7A, 7B and S10B). Similarly, cells not killed by β-estradiol treatment also became more differentiated (Figures 7A, 7B and S10B). Interestingly, MAPK inhibition and β-estradiol treatment altered the cellular phenotype by a similar extent (Figure 7B). RA is used clinically to differentiate neuroblastoma but is not as effective for MNA tumours as MYCN single copy ones [52]. We therefore compared the level of differentiation achieved by RA with CI-1040 and β-estradiol treatment. CI-1040 and β-estradiol induced a level of differentiation on a par with RA (Figures 7A, 7B and S10B). Combination treatment with RA and CI-1040 did not increase the level of differentiation obtained beyond that of RA only treatment (Figure 7B). However, the β-estradiol and RA combination significantly enhanced the level of differentiation obtained, compared with single-agent treatment (Figure 7B, t-test: p<0.0001). Although none of the treatments fully differentiated IMR32 cells, a propensity to make any cells not killed by CI-1040 or β-estradiol more differentiated and less stem-like would still be an added benefit of such therapy.

We next assessed the effect of co-treatment on cell viability. RA had little effect on the viability of IMR32 cells. However, upon combination treatment RA further sensitised the cells to MAPK (MEK1/2) inhibition (CI-1040) and β-estradiol. The combination treatment more than doubled the loss of viability over that seen in CI-1040 only treated cells (Figure 7C). Taken together, these results show that both CI-1040 and β-estradiol hold potential as therapeutic agents for MNA neuroblastoma, both as single agents and in combination with other clinically utilised therapeutics, such as RA. The majority of MNA neuroblastoma cells were killed by these compounds, with surviving cells being more differentiated in appearance.

## DISCUSSION

### MYCN globally supresses cellular networks and re-wires its own interactome

Our data suggest that MYCN, like c-MYC [53, 54], has both transcriptional activator and repressor functions. This recently re-asserted view [53, 54] qualifies the previously reported function of c-MYC as a general transcriptional enhancer that only amplifies the expression of already activated genes [55, 56]. We show that MYCN-regulated transcription differs depending on whether MYCN was amplified (predominantly activated) or overexpressed (predominantly repressed). Despite this, both overexpressed and amplified MYCN mainly suppressed the activity of transcriptional regulator networks. Oncogenic MYCN therefore achieves its harmful effects primarily through the suppression of downstream effector networks.

Interestingly, MYCN transcriptionally regulates the expression of many of its protein interaction partners, thus altering the landscape and functionality of the MYCN interactome. Amplified MYCN was a more promiscuous binder than overexpressed MYCN, having many more binding partners, even when cell lines with similar protein levels of overexpressed or amplified MYCN are compared (Figure S3). In addition, other factors that contribute to MNA-driven neuroblastoma were recently identified. They include the co-amplification of MYCN with lncUSMycN, a long non-coding RNA upstream of the MYCN locus, and NCYM, a protein transcribed from the anti-sense strand that inhibits GSK3β [57, 58]. Combined, these findings help explain the differing prognosis of neuroblastoma patients with elevated or amplified MYCN [7-10]. The re-wiring of its own interactome, saturation of MAX and suppression of cellular networks likely contributes to aberrant MYCN function, driving the divergent phenotypes of normal and oncogenic MYCN signalling.

### Identification of targetable vulnerabilities with therapeutic potential

Our study also demonstrates that applying an integrated omics approach, rather than using single omic technologies in isolation, facilitates rapid cross-platform validation and is capable of elucidating disease-relevant molecular mechanisms. Furthermore, analysis of the omics data at a systems level as opposed to gene level enables easier integration, overcoming complications arising from each individual platform. Such analysis accurately revealed disease-relevant networks and highlighted therapeutically targetable vulnerable nodes. Crucially, we validated a network-based approach to overcome the limitations of classical genetic approaches to therapeutic target identification in complex multifactorial diseases, such as neuroblastoma, which lack frequently altered genetic drivers [27].

Our MYCN integrative omic analysis revealed a host of known genes, pathways and drugs that were previously associated with neuroblastoma, including p53, TGFβ, TNF, IFNG, Retinoic Acid and epigenetic modifiers [12, 22, 24, 48, 49]. While these confirmatory findings bolster confidence in the analysis, our data also provide a wealth of information regarding potential novel therapeutic targets for MNA neuroblastoma, ranging from epigenetic regulators to classical signalling pathway components. Mapping the upstream regulator networks that drive DE genes in highly MNA cells compared with MYCN single copy cells elucidated several central nodes not previously linked to MNA neuroblastoma (e.g. HRAS, MAPK1, RAF1, NEUROG1 and ER).

MAPK was the most enriched pathway in an analysis of ITRs common to the metastatic MNA cell lines. The MAPK1 gene was bound by MYCN, and MAPK1 became progressively more activated with increasing levels of MYCN amplification. There is an emerging role of MAPK signalling in neuroblastoma [59-61], including the occurrence of MAPK pathway mutations in relapsed tumours [62-65]. Here, we show that MYCN interacts with the MAPK pathway at numerous levels, including through protein-protein interactions with component proteins of the pathway, revealing that MAPK can play a role in neuroblastoma even before MAPK mutations occur. Furthermore, we found that neuroblastoma cells are susceptible to MEK inhibition, which could prove to be therapeutically beneficial as potent MEK inhibitors are available for clinical use [66], with CI-1040 itself being orally active [67]. Additionally, we revealed that co-treatment of RA and CI-1040 can further sensitise metastatic MNA cells to MAPK inhibition, more than doubling the observed loss of viability.

Like CI-1040, orally active forms of β-estradiol exist, with β-estradiol being clinically approved for a range of conditions, and in advanced clinical trials (stage 3 and 4) for many others, including breast and prostate cancer (IPA database and www.clinicaltrials.gov). MNA cells were preferentially affected by β-estradiol treatment, which indicates potential utility as a targeted therapy. To this end we also report a gene signature capable of stratifying patients based on their β-estradiol activity status.

Interestingly, both β-estradiol and Estrogen receptor signalling were identified as ITRs of the MNA cells (compared with MYCN single-copy cells), but they showed an inverse activation status. β-estradiol was inhibited in MYCN amplified cells, whereas Estrogen receptor signalling (estrogen receptors are the primary receptor of β-estradiol) was activated. Similarly, β-estradiol was activated upon induction of differentiation, whereas estrogen receptor signalling was inhibited. This apparent contradiction is resolved by the fact that β-estradiol can bind a number of other proteins including AR, SHBG, NCOA3, NCOA2, Ste, CYP3A4, Iebp, AFP, ERBB2, GST A1-1 and NR0B1 (IPA database). Indeed, the target genes contributing to the identification of the ERs and β-estradiol ITR were largely different reflecting the fact that β-estradiol likely alters neuroblastoma cell fate mainly independently of its estrogen receptor functions. Estrogen receptor activation has recently been linked to increased proliferation, migration and invasion of MYCN single-copy SY5Y cells [68]. We observed no loss of viability when MYCN single-copy cells were treated with β-estradiol. However, our results demonstrate the specific anti-oncogenic potential of β-estradiol in MYCN amplified neuroblastoma, where it reduces cell viability and partially removes the MYCN mediated differentiation block.

Given the high degree of connectedness between the novel and already established therapeutically targetable nodes our analysis identified, opportunities are likely to exist for exploiting synergies by simultaneously targeting multiple vulnerabilities. We demonstrated the benefit of such combination approaches by simultaneously activating RA signalling and either treating with β-estradiol or inhibiting MAPK signalling. Targeting vulnerabilities in the amplified MYCN regulatory network not only reduced cell viability but also impaired MYCN's ability to inhibit differentiation. While targeting a single node did not fully remove IMR32's resistance to RA mediated differentiation, co-treatments were synergistic at reducing overall cell viability. Therefore, CI-1040 or β-estradiol combination treatments with RA would likely enhance the therapeutic potential of RA in MNA tumours.

The relevance of the network parameters obtained from the cell line omics data to patient outcome was verified both in terms of predicting correlations in expression between genes and by segregating patient outcome. This provides further confidence that our results reflect the true underlying biology of MYCN and are applicable to the clinical setting. Our analysis approach not only identified potential drug targets but simultaneously provided a predictive gene signature to inform personalised treatment strategies. Aside from new targets this approach could also potentially be used to generate stratification signatures for already clinically validated treatments with heterogeneous patient responses.

## CONCLUSIONS

Oncogenic MYCN re-wires its own interaction and signalling networks to repress regulators of normal cellular developmental processes, such as differentiation. By reconstructing the MYCN network we identified novel therapeutically targetable vulnerabilities. Finally, our data show that an integrative omics analysis can reveal molecular pathogenic mechanisms of multifactorial disease and new targets for therapy, and enable a systems medicine approach that stratifies and eventually will treat patients based on multivariate molecular data.

## MATERIALS AND METHODS

### Cell culture & treatments

For cell culture conditions and cell sources see Duffy et al. [69]. MYCN overexpression in the SH-SY5Y/6TR(EU)/pTrex-Dest-30/MYCN (SY5Y-MYCN) [69, 70] line was induced by 1μg/ml Doxycycline (Sigma). SY5Y-MYCN media was supplemented with 7.5μg/ml Blasticidine (Sigma) and 200μg/ml G418 (Sigma). Small molecules used were: CI-1040 (PD184352, Sigma), β-estradiol (Sigma) and Retinoic Acid (RA, Sigma). Stock solutions were dissolved in DMSO, except for β-estradiol for which ethanol was used. Compounds were replenished every 24h for any treatment longer than a 24h duration.

### Western blot & RT-qPCR

Western blotting and RT-qPCR were performed as previously described [69]. Antibodies used were MYCN (1/1,000 dilution, sc-53993, Santa Cruz) and Tubulin (1:1000, Santa Cruz, Cat No. sc-8035). For TaqMan assays and primer sequences, used with TaqMan and SYBR reagents respectively, (Applied Biosystems) see Table S7.

### Deep sequencing and bioinformatics analysis (mRNA-, miRNA- and ChIP-seq) and patient tumour microarrays

RNA-seq was conducted and subsequent bioinformatics analysis performed as previously described [69], for 5 neuroblastoma cell lines and the MYCN over expression time-course (un-induced, 1h, 4h and 24h). In addition to standard mRNA-seq, 4sU labelling mRNA-seq was performed on some of the time-course samples (un-induced, 1h and 4h). These samples were incubated in 500μM 4-thiouridine (4sU, Sigma) 30 minutes before cell lysis, to label only the mRNAs synthesised during that time. For an in-depth description of the 4sU-seq experiment and associated bioinformatics analysis see Schwarzl et al. 2015 [31]. Each sample was separated into 3 pools, total, labelled and un-labelled (pre-existing) as previously described [71, 72]. Additional samples (un-induced and 48h) were sequenced with a single read run (TruSeq SR cluster kit v5, Illumina). Non-coding RNAs were identified using the Reference Annotation Based Transcript (RABT) option in Cufflinks. miRNA libraries (SY5Y-MYCN; un-induced, 1h, 4h and 24h MYCN induction) were prepared using TruSeq small RNA sample preparation kit (Illumina) and sequenced as per mRNA (single read). miRNAs were called with miRanalyser [73] (http://bioinfo5.ugr.es/miRanalyzer/miRanalyzer.php). All RNA-seq samples were performed in biological duplicate and sequenced on an Illumina GAIIx.

ChIP-seq was performed on KCN, KCNR and SY5Y-MYCN (un-induced, 24h and 48h MYCN induction) cells following the SimpleChIP Plus Enzymatic Chromatin IP Agarose Beads Kit (Cell Signalling Technology) protocol using the MYCN antibody (as above) or anti-Mouse IgG (M5899, Sigma), libraries were generated using NEXTflex ChIP-Seq Kit (Bioo Scientific) and sequenced as above (single read). Data formatting and peak calling (MACS V1.0.1) were performed using Galaxy (www.usegalaxy.org). Peaks were visualised using SeqMonk v29.0.0 (http://www.bioinformatics.babraham.ac.uk/projects/seqmonk/). All sequencing samples were performed in biological duplicate and sequenced on an Illumina GAIIx or HiSeq 2500. MYCN's genomic targets were analysed for known regulatory elements and binding motifs using the DiRE [35] and SIOMICS [34] tools respectively.

### Additional software tools

Ingenuity Pathway Analysis (IPA) software was also used for the ITR, pathway and gene ontology (GO) analysis. String (www.string-db.org) was used to generate protein-protein interaction networks, and the KEGG pathway enrichment analysis tool in Sting was also applied to these networks. Area-proportional Venn diagrams were generated using BioVenn (www.cmbi.ru.nl/cdd/biovenn) and four-way comparisons were generated using Venny (http://bioinfogp.cnb.csic.es/tools/venny/index.html). Measurements of neurite length and cell width were obtained from images using ImageJ v1.44p (http://imagej.nih.gov/ij).

### Patient microarray data

Sample set composition, sample preparation and generation of single-color gene-expression profiles from primary neuroblastoma were described previously [38]. Raw and normalized microarray data are available in ArrayExpress database (E-TABM-38, E- MTAB-161, E-MTAB-179).

### Proteomics

Mass spectrometry based interaction proteomics were conducted on SY5Y-MYCN (un-induced, 4h, 24h and 48h) and KCN as previously described [74], for the MYCN protein. MYCN was immunoprecipitated by using Protein A/G PLUS-agarose beads (sc-2003, Santa Cruz) conjugated to MYCN antibody or IgG (as above). Three biological and two technical replicates were performed per condition.

### Cell viability and proliferation assay

Cell viability was analysed by MTS assay as described [69], with values normalised to the control of the same day.

### High-throughput RNAi screens

SY5Y-MYCN induced and un-induced cells were used for the RNAi screen. Before the high-throughput screening, cell number was titrated to ensure that cell proliferation remained in a linear exponential phase throughout the experiment. The siRNA library targeting human druggable genome (Qiagen GmbH, Germany) consists of four siRNAs per gene. These four siRNAs per gene were pooled into the same well (final assay concentration 50nM) on 384-well white, clear-bottom assay plates (Greiner Bio-One GmbH, Frickenhausen, Germany), followed by addition of siLentFect (Bio-Rad Laboratories, Hercules, CA) using a Multidrop 384 Microplate Dispenser (Thermo Fisher Scientific Inc, Waltham, MA, USA) for 1h at room temperature. Cell suspension (1500 cells per well) was thereafter overlaid and the plates were incubated for 72 h at +37°C. Cell viability was measured using CellTiter-Glo Luminescence Assay (Promega, Madison, WI, USA) with Envision Plate-reader (PerkinElmer/Wallac) according to manufacturer's instructions. The raw data were normalized using “loess-samp” normalization method, implemented in R (R Core Team, 2014 http://www.r-project.org/), omitting plate rows 1-2 and 23-24 from normalisation which contain controls, and thereby normalizing the “sample fraction” of the plate, only. After loess correction, z-scores were calculated using robust estimators median and median absolute deviation of the distribution of the corrected values.

### Accession codes

Sequencing data were deposited in ArrayExpress (www.ebi.ac.uk/arrayexpress) under accession numbers, E-MTAB-2690, E-MTAB-2691, E-MTAB-1684, E-MTAB-2787, E-MTAB-4100 and E-MTAB-2689.

## SUPPLEMENTARY MATERIAL FIGURES AND TABLES

















## Abbreviations

MNA: MYCN amplified
ITR: inferred transcriptional regulator
CPMkb: read counts per million adjusted by gene length in kilobases
DE: differentially expressed
TSS: transcription start sites
IPA: Ingenuity pathway analysis
ER: estrogen receptor
RA: Retinoic Acid
FC: fold change
ChIP: chromatin-immunoprecipitation
coIP: co-immunoprecipitation
