# Supplementary material for: Integrative omics reveals MYCN as a global suppressor of cellular signalling and enables network-based therapeutic target discovery in neuroblastoma
Source: Oncotarget. 2015 Dec 11;6(41):43182–201. doi: 10.18632/oncotarget.6568 (PMC4791225; doi:10.18632/oncotarget.6568)
Supplement: Supplementary file 1 [file oncotarget-06-43182-s001.pdf]

# Integrative omics reveals MYCN as a global suppressor of cellular signalling and enables network-based therapeutic target discovery in neuroblastoma

**Supplementary Material**

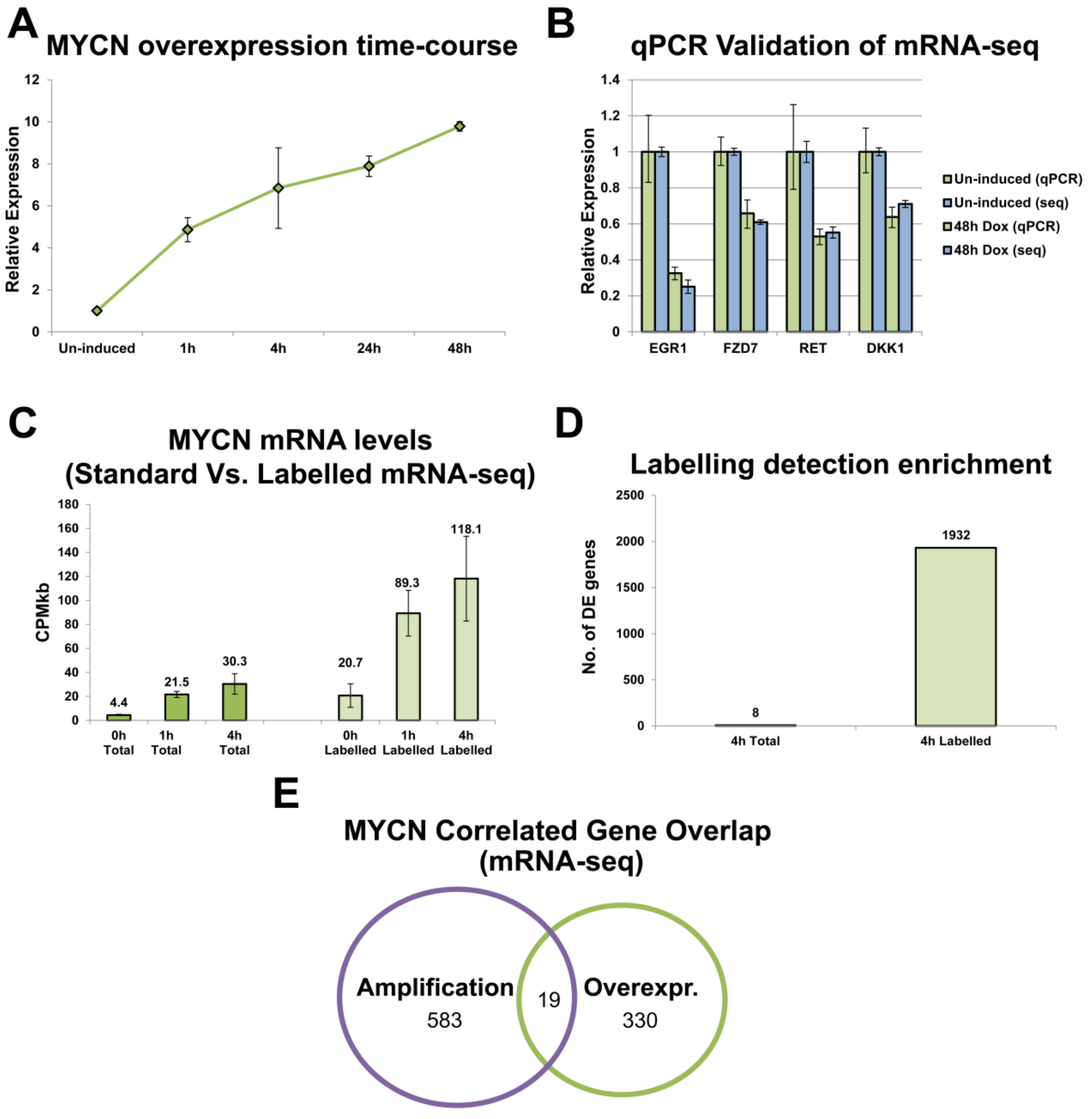


**Figure S1. Additional mRNA-seq data from MYCN overexpressing cells. (A)** MYCN mRNA relative expression across the SY5Y-MYCN overexpression time-course. Data from

mRNA-seq total samples. **(B)** Validation of selected SY5Y-MYCN mRNA-seq results by qPCR. Expression is relative to the expression level in the control samples. **(C)** MYCN mRNA expression levels, as detected by standard (Total mRNA) mRNA-seq and 4sU labelled (Labelled mRNA) mRNA-seq. 4sU labelling increased the number of counts for actively transcribed genes. **(D)** 4sU labelling (Labelled) mRNA-seq greatly enriched the number of differentially expressed (DE) genes detectable at 4h post MYCN induction, compared to standard mRNA-seq (Total). **(E)** Overlap of MYCN correlated genes, shown in Fig. 1D, MNA (IMR32, Kelly, KCN and KCNR) versus overexpressed (SY5Y-MYCN time-course).

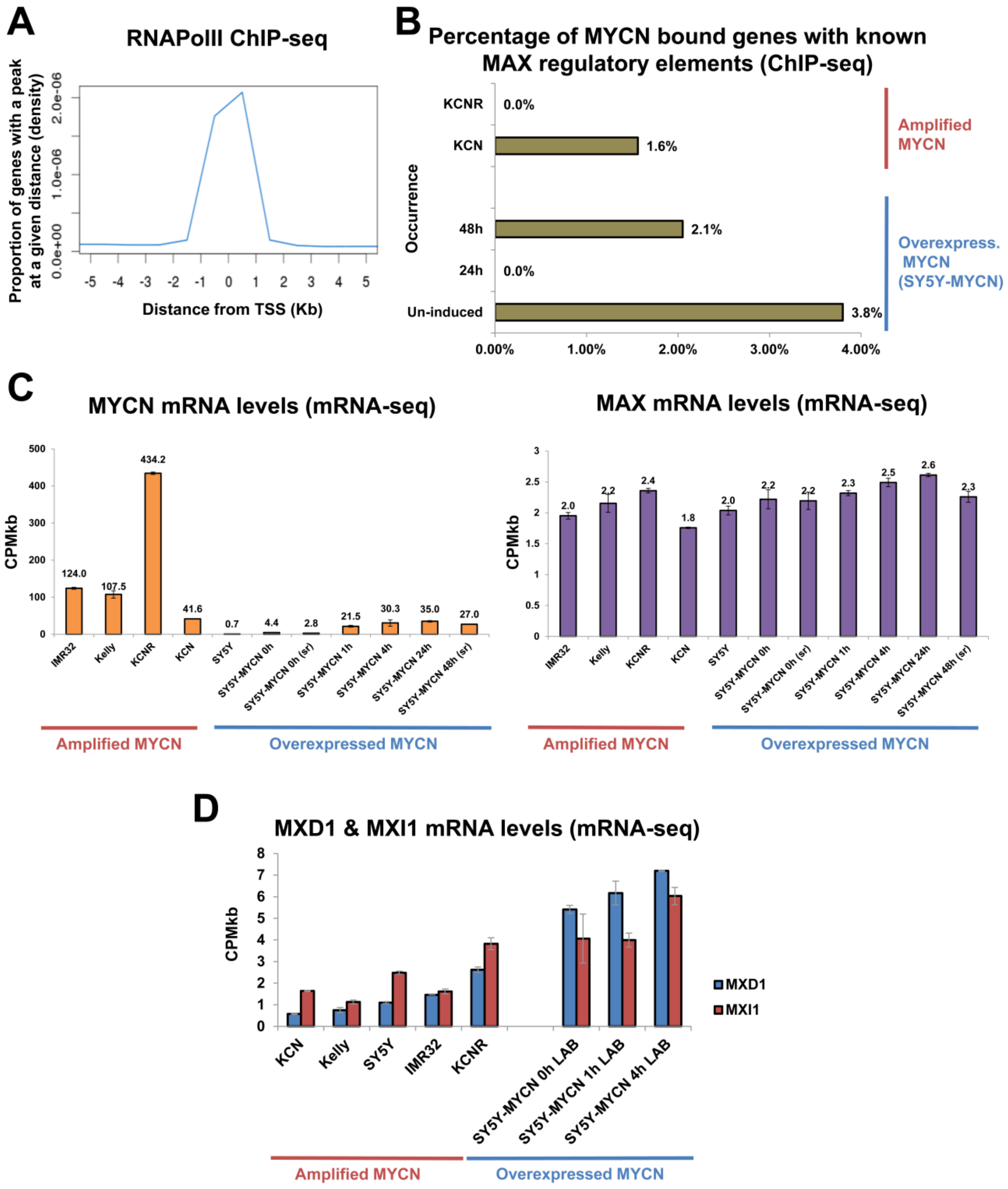


**Figure S2. Additional ChIP-seq and mRNA expression data for MYCN, MAX, MXD1 and MXI1.** **(A)** RNApolII ChIP-seq binding peak profile in SY5Y-MYCN cells induced with Doxycycline for 48h. +/-5kb around the TSS shown. **(B)** DiRE analysis (http://dire.dcode.org) showing the percentage (occurrence) of MYCN bound genes from the MYCN ChIP-seq data

(KCN, KCNR and SY5Y-MYCN cells) which are also MAX targets, i.e. known to have MAX binding regulatory elements. **(C)** MYCN (left) and MAX (right) mRNA expression across the mRNA-seq samples. (sr) denotes single read sequencing run, the other samples are paired-end runs. **(D)** MXD1 and MXI1 mRNA expression across the mRNA-seq samples. 4sU labelled; LAB, with the rest being standard mRNA-seq (Total).

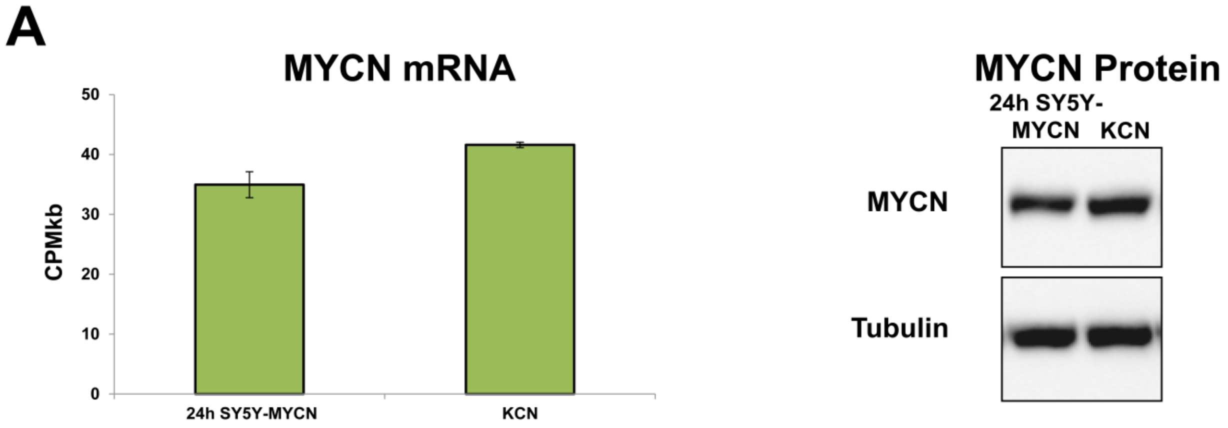


**Figure S3. Additional MYCN expression level data. (A)** Comparison of MYCN mRNA (left) and protein (right) expression levels in 24h induced SY5Y-MYCN and KCN cells, as measured by RNA-seq and Western blot respectively.

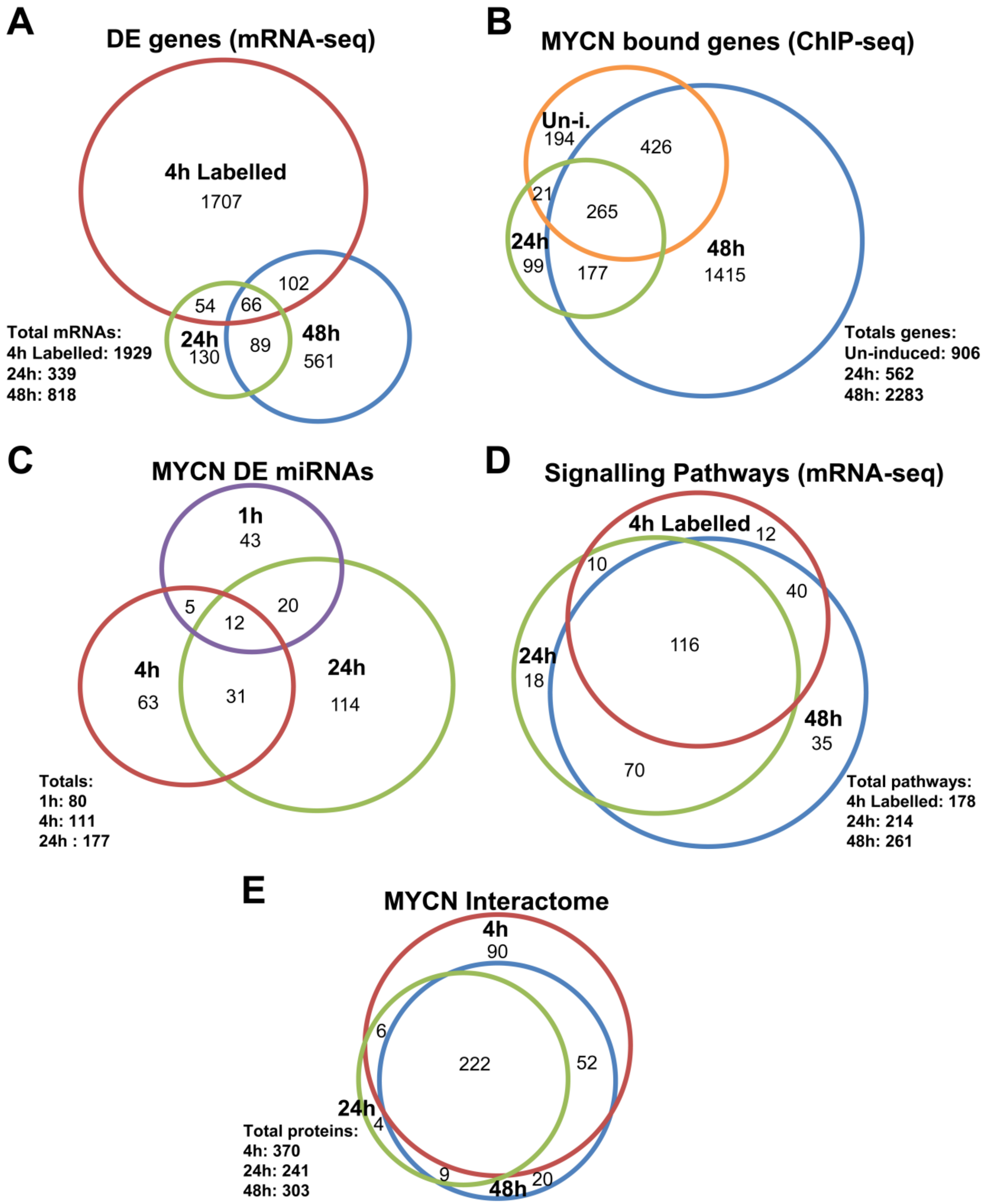
A
DE genes (mRNA-seq)
4h Labelled
1707
102
54
66
24h
130
89
48h
561
Total mRNAs:
4h Labelled: 1929
24h: 339
48h: 818
B
MYCN bound genes (ChIP-seq)
Un-i.
194
426
21
265
24h
99
177
48h
1415
Totals genes:
Un-induced: 906
24h: 562
48h: 2283
C
MYCN DE miRNAs
1h
43
5
20
12
4h
63
31
24h
114
Totals:
1h: 80
4h: 111
24h : 177
D
Signalling Pathways (mRNA-seq)
4h Labelled
12
10
40
116
24h
18
48h
35
70
Total pathways:
4h Labelled: 178
24h: 214
48h: 261
E
MYCN Interactome
4h
90
6
222
52
24h
4
9
48h
20
Total proteins:
4h: 370
24h: 241
48h: 303

**Figure S4. Temporal expression of overexpressed MYCN targets and temporal conservation of signalling pathways (SY5Y-MYCN), identifying a level for integration.** All data were generated in SY5Y-MYCN cells **(A)** Overlap of differentially expressed (DE) genes identified by mRNA-seq upon MYCN overexpression. DE genes called based on comparison with the relevant un-induced control samples. **(B)** Overlap of genes (nearest coding gene to DNA region bound by MYCN protein, identified by MYCN ChIP-seq. **(C)** Overlap of differentially expressed (DE) miRNAs identified by miRNA-seq upon MYCN overexpression. DE miRNAs called based on comparison with the un-induced control samples. **(D)** Overlap of signalling pathways (identified by IPA) whose components are differentially regulated upon MYCN overexpression (mRNA-seq). **(E)** Overlap of proteins which bound to MYCN protein (MYCN coIP) upon induction of MYCN overexpression (SY5Y-MYCN).

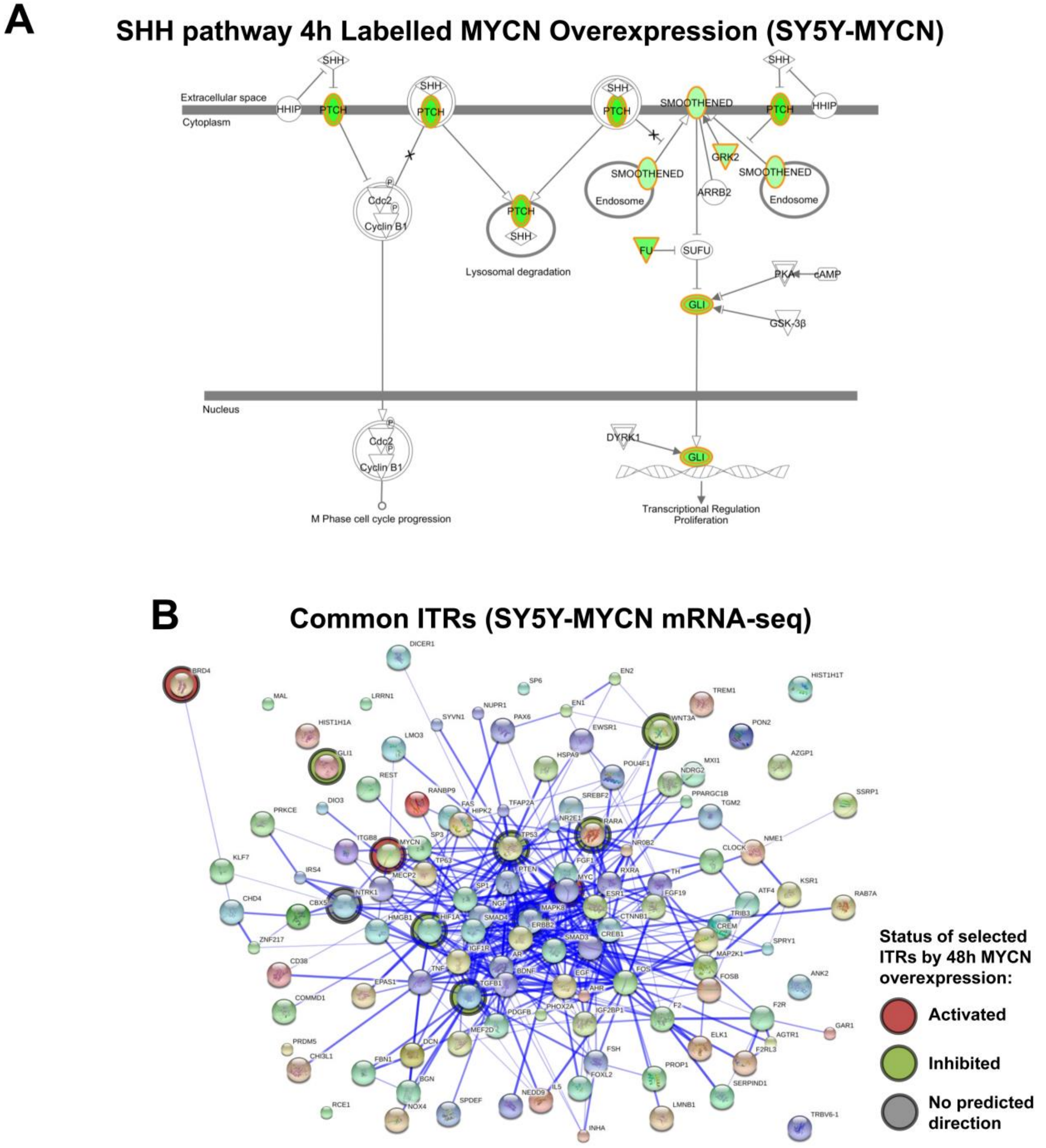


**Figure S5. Additional pathway- and network-level analysis of overexpressed MYCN (SY5Y-MYCN) (A)** Sonic Hedgehog (SHH) Signalling pathway components differentially regulated at 4h MYCN overexpression (4sU labelled mRNA-seq), generated with IPA. Downregulated SHH signalling pathway components are shaded green, no components were upregulated. DE components on the schematic which are single genes have a single orange outline, while components representing more than one gene (gene families) have a double orange

outline. **(B)** ITRs common to at least two mRNA-seq time-points were used to build a protein-protein interaction map based on previously known interactions from the String database. Width of connecting lines indicate the confidence of evidence. Activation status, at 48h of MYCN overexpression, of selected ITRs (mentioned in the text) are circled, red for activated and green for inhibited ITRs.

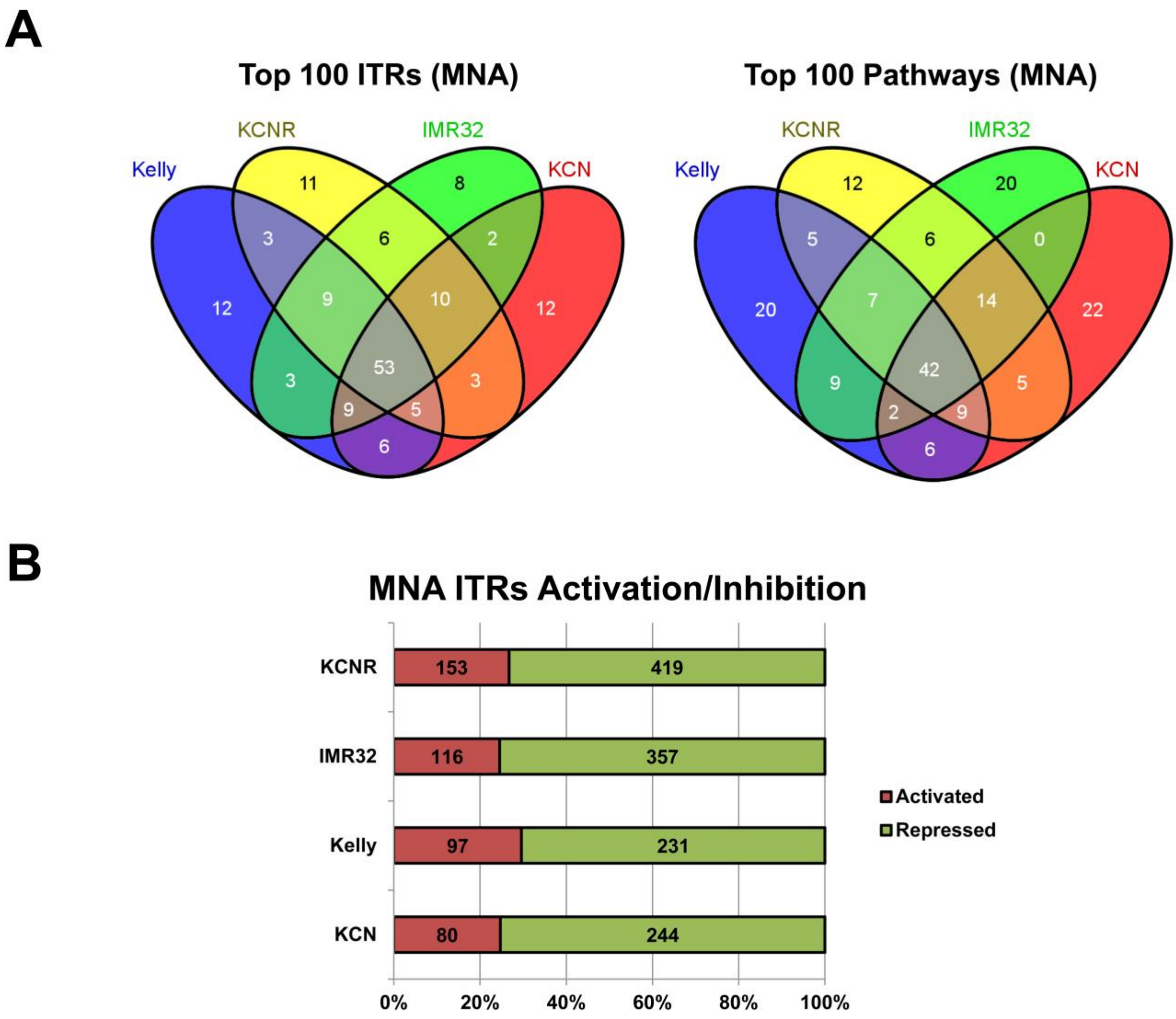


**Figure S6. Additional MNA pathway and ITR analysis. (A)** Overlaps between top 100 ITRs (left) and differentially expressed pathways (right) in MNA lines compared with SY5Y. ITRs and pathways were identified by IPA analysis. **(B)** Proportions of activated and inhibited ITRs based on the identified DE genes (mRNA-seq) in each of the MNA cell lines. SY5Y cells, which are MYCN single copy, were used as the control cell line. Absolute numbers of activated and repressed ITRs are shown within each bar.

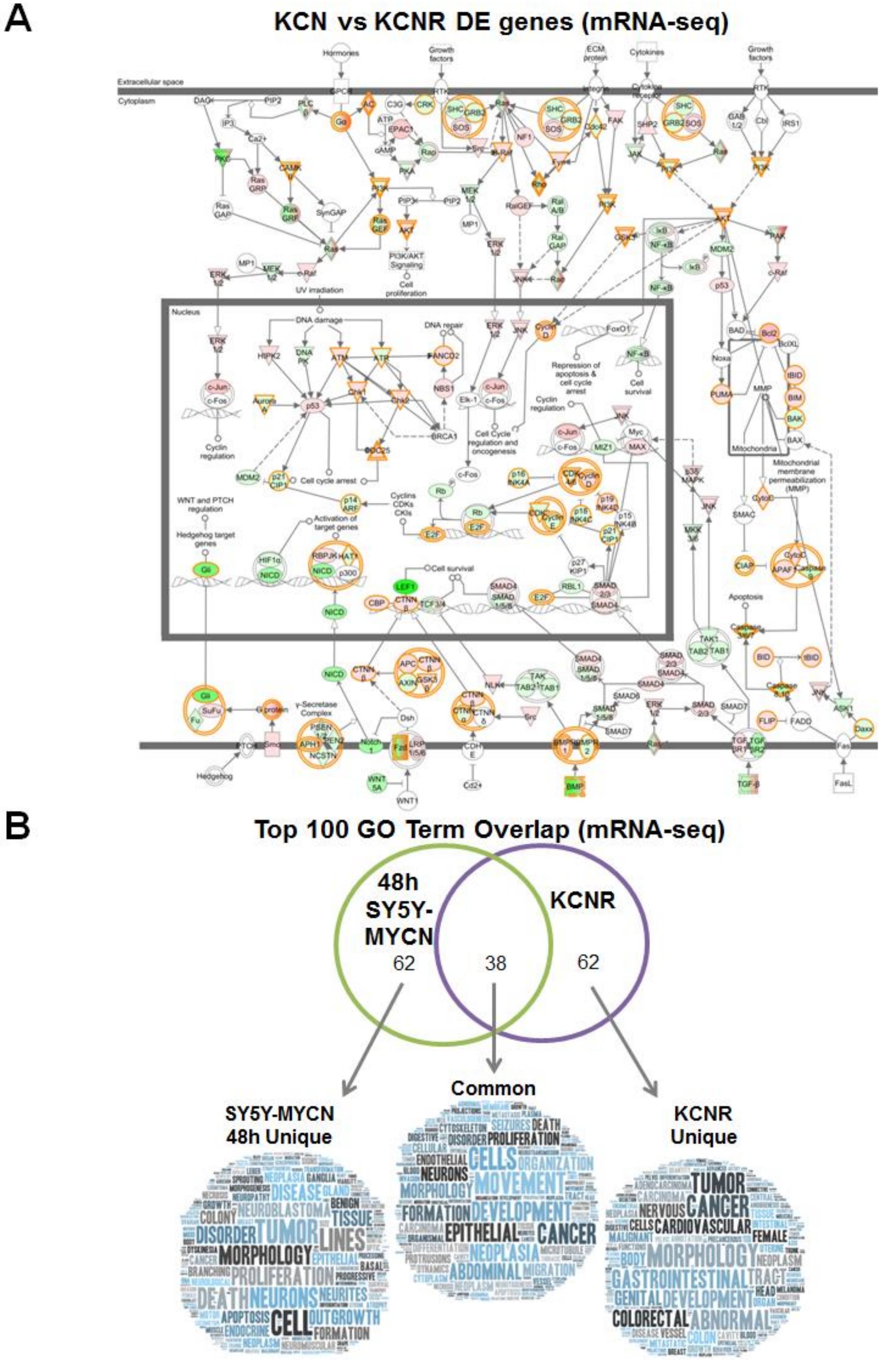
A
KCN vs KCNR DE genes (mRNA-seq)
B
Top 100 GO Term Overlap (mRNA-seq)
48h SY5Y-MYCN
KCNR
62
38
62
SY5Y-MYCN 48h Unique
Common
KCNR Unique

**Figure S7. Differentially expressed oncogenic network components between KCN and KCNR patient matched cell lines, and global comparison of MYCN amplified and overexpressed GO terms. (A)** Network reconstruction based on mRNAs differentially expressed between patient matched KCN (primary) and KCNR (metastatic) cell lines, generated using IPA. Red shaded genes are upregulated in KCNR, while green ones are downregulated. **(B)** Overlap of the top 100 GO terms (IPA) for 48h MYCN overexpressing SY5Y-MYCN and MNA KCNR (top). GO terms from each of the 3 groups (48h SY5Y-MYCN unique, KCNR unique and common to both) were used to generate weighted images (bottom) with Tagxedo (www.tagxedo.com). The more frequently a word appeared on the GO term list the larger it is in the image. Note that GO terms were split into their individual component words for the weighted image analysis.

**A**

**MYCN Overexpression MAPK ITRs (RNA-seq)**

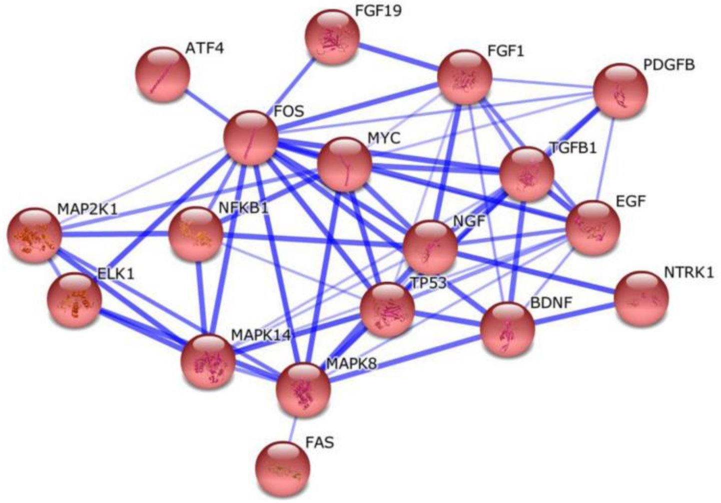


**B**

**MAPK signalling pathway components with amplified MYCN protein-protein interactions (KCN co-IP Mass. Spec.)**

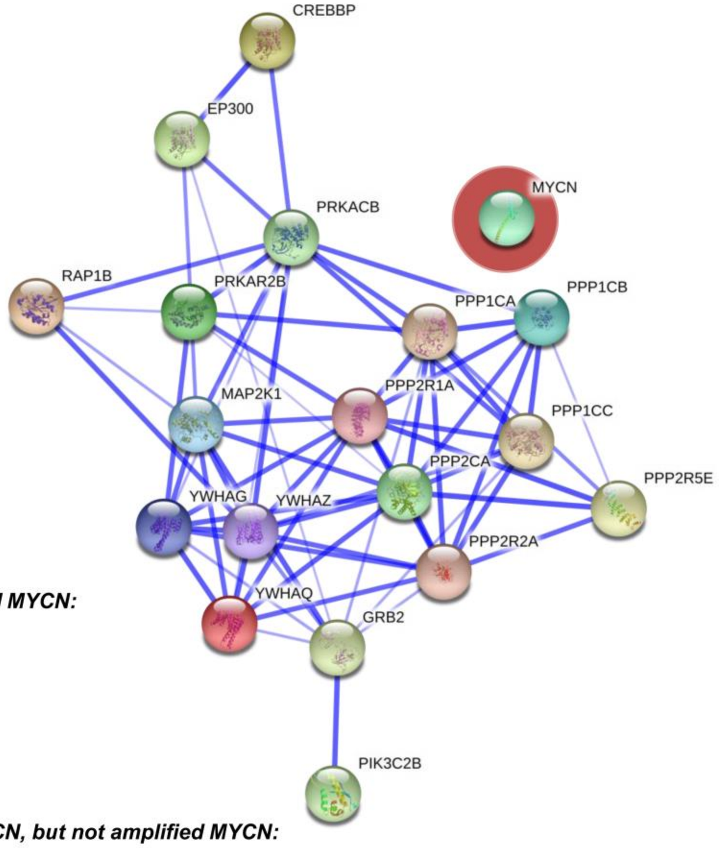


***Also bound by 24h overexpressed MYCN:***

GRB2
PPP2CA
PPP2R1A
PPP2R2A
PPP2R5E
RAP1B
YWHAQ

***Bound by 24h overexpressed MYCN, but not amplified MYCN:***

PPP1R10
RRAS2

**Figure S8. Additional MAPK ITR and interactome data. (A)** Protein-protein interaction network of MAPK associated (identified by KEGG) ITRs which were common to at least 2 time-points of the MYCN overexpression time-course. For full network, including non-MAPK components, see Fig. 5D. **(B)** Protein-protein interaction network of components of the MAPK signalling pathway which were found to interact with amplified MYCN protein in the KCN cell line. MYCN is highlighted by a red circle and has no previously known interactions with these proteins (String database). A list of components of the MAPK signalling pathway which were bound by overexpressed MYCN at 24h of induction (SY5Y-MYCN) is inset.

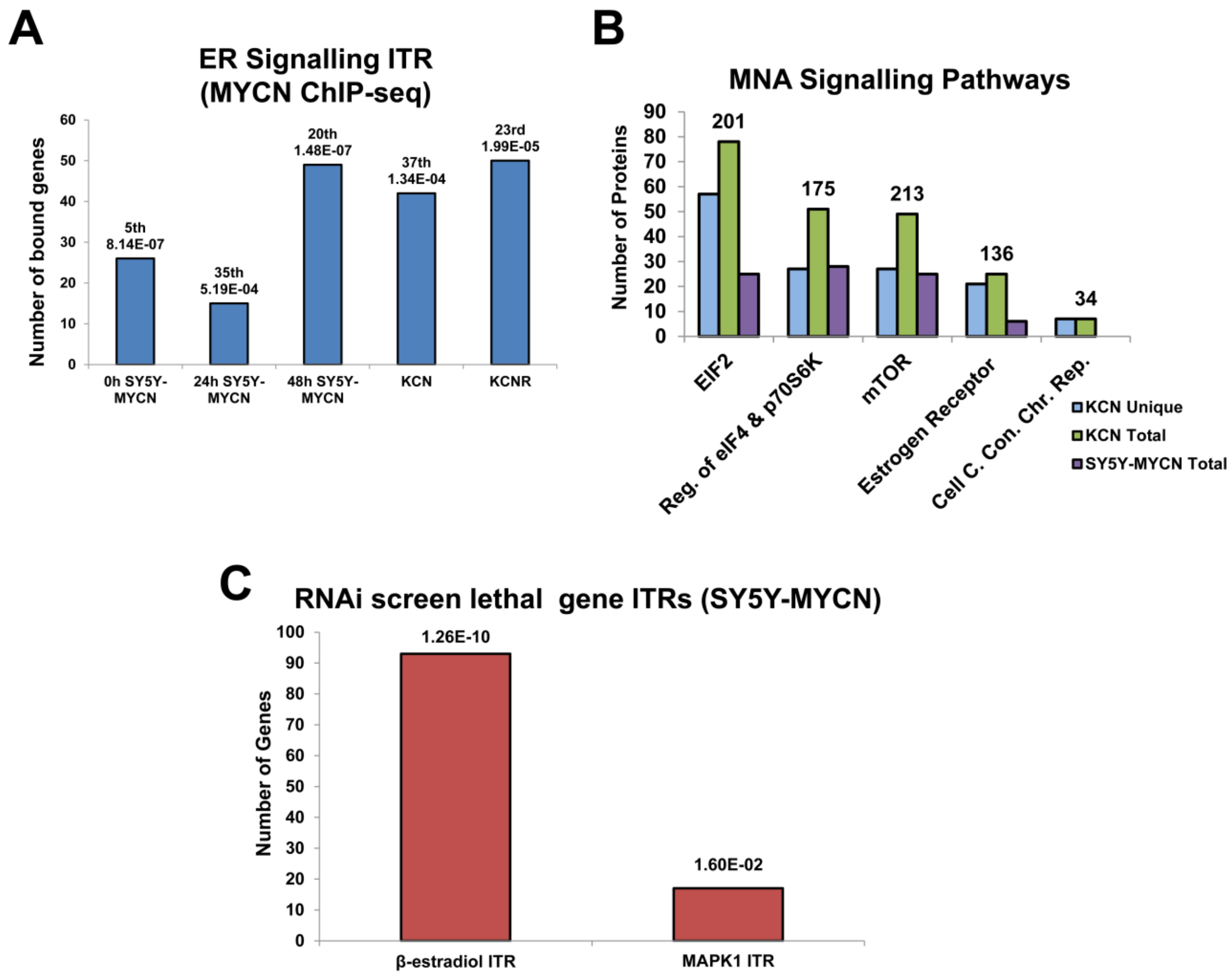


**Figure S9. Additional Estrogen Receptor Signalling data. (A)** Estrogen receptor signalling was identified as an ITR of all 3 MYCN ChIP-seq time-points in SY5Y-MYCN and in KCN and KCNR cells. Number of ER signalling genes which were bound by MYCN protein (ChIP-seq) are shown. **(B)** Top 5 signalling pathways (IPA) associated with the proteins which bound amplified (KCN) but not overexpressed MYCN (24h SY5Y-MYCN), as identified by MYCN coIP mass spectrometry. 'Total' denotes all MYCN bound proteins detected. 'Unique' denotes only the fraction of proteins which bound MYCN in KCN but not SY5Y-MYCN. The absolute number of known proteins in each pathway is shown above the bars, while the y-axis shows the number of these that were detected as being bound to MYCN. The $5^{th}$ pathway's full name is Cell Cycle Control of Chromosomal Replication. **(C)** Numbers of β-estradiol and MAPK1 target

genes, as identified by IPA ITR analysis, which strongly (greater than 2 standard deviations from the median of the screen) reduced SY5Y-MYCN viability (in either condition) when knocked down (RNAi screen with druggable-genome siRNA library).The p-value of overlap (between the RNAi hits corresponding to the ITR and all known target genes of that ITR) shown above each bar.

**A**

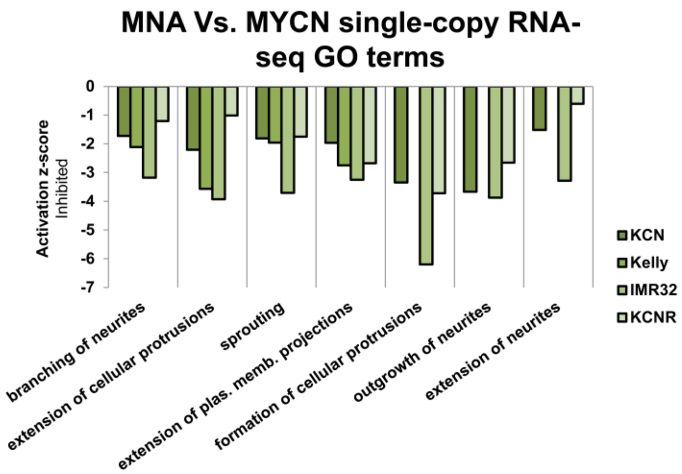


**B**

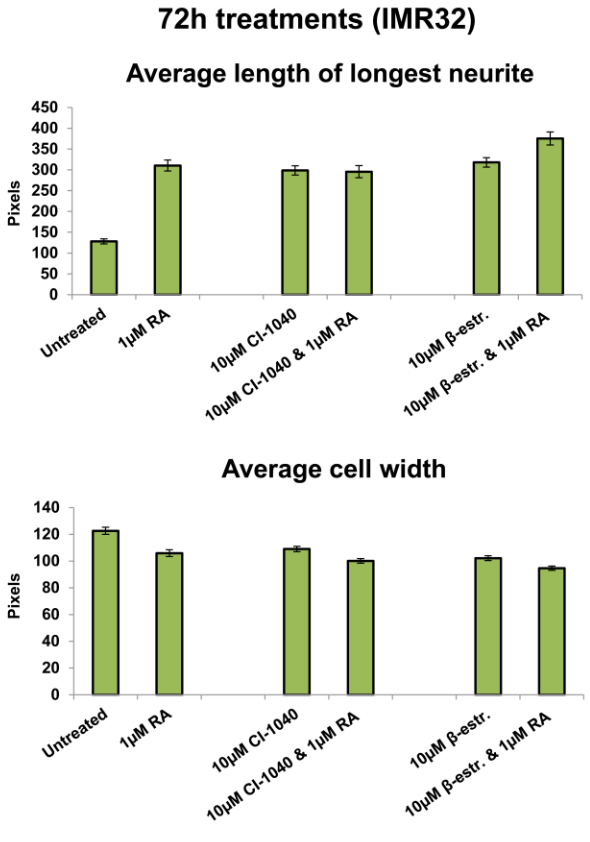


**Figure S10. Additional differentiation data. (A)** IPA disease and function GO term analysis of RNA-seq samples revealed that genes differentially expressed (cut-offs: >1log$_2$FC and p<0.01) between MYCN amplified (KCN, Kelly, IMR32 and KCNR) and MYCN single-copy (SY5Y) cells lines were enriched for genes associated with the inhibition of neuritogenesis. **(B)** The

average length of the longest neurite (top) and cell width (bottom) in IMR32 cells treated for 72h with individual agents or combination treatments with RA. Range of measured cells (N) per treatment group is 132-209. Error bars depict the standard error of the mean. Measurements made using ImageJ v1.44p (http://imagej.nih.gov/ij ).
